# A Genomic Screen Revealing the Importance of Vesicular Trafficking Pathways in Genome Maintenance and Protection against Genotoxic Stress in Diploid *Saccharomyces cerevisiae* Cells

**DOI:** 10.1371/journal.pone.0120702

**Published:** 2015-03-10

**Authors:** Kamil Krol, Izabela Brozda, Marek Skoneczny, Maria Bretne, Adrianna Skoneczna

**Affiliations:** 1 Laboratory of Mutagenesis and DNA Repair, Institute of Biochemistry and Biophysics Polish Academy of Sciences, Warsaw, Poland; 2 Department of Genetics, Institute of Biochemistry and Biophysics Polish Academy of Sciences, Warsaw, Poland; 3 Faculty of Chemistry, Warsaw University of Technology, Warsaw, Poland; The University of Hong Kong, HONG KONG

## Abstract

The ability to survive stressful conditions is important for every living cell. Certain stresses not only affect the current well-being of cells but may also have far-reaching consequences. Uncurbed oxidative stress can cause DNA damage and decrease cell survival and/or increase mutation rates, and certain substances that generate oxidative damage in the cell mainly act on DNA. Radiomimetic zeocin causes oxidative damage in DNA, predominantly by inducing single- or double-strand breaks. Such lesions can lead to chromosomal rearrangements, especially in diploid cells, in which the two sets of chromosomes facilitate excessive and deleterious recombination. In a global screen for zeocin-oversensitive mutants, we selected 133 genes whose deletion reduces the survival of zeocin-treated diploid *Saccharomyces cerevisiae* cells. The screen revealed numerous genes associated with stress responses, DNA repair genes, cell cycle progression genes, and chromatin remodeling genes. Notably, the screen also demonstrated the involvement of the vesicular trafficking system in cellular protection against DNA damage. The analyses indicated the importance of vesicular system integrity in various pathways of cellular protection from zeocin-dependent damage, including detoxification and a direct or transitional role in genome maintenance processes that remains unclear. The data showed that deleting genes involved in vesicular trafficking may lead to Rad52 focus accumulation and changes in total DNA content or even cell ploidy alterations, and such deletions may preclude proper DNA repair after zeocin treatment. We postulate that functional vesicular transport is crucial for sustaining an integral genome. We believe that the identification of numerous new genes implicated in genome restoration after genotoxic oxidative stress combined with the detected link between vesicular trafficking and genome integrity will reveal novel molecular processes involved in genome stability in diploid cells.

## Introduction

Living cells have developed various mechanisms to detect and repair damage that occurs under stress conditions. One of the macromolecules that can be affected by oxidative stress is DNA, and the types of damage may be diverse. Such types of damage include single- and double-strand breaks (SSBs and DSBs), which are difficult to repair. DNA breaks are dangerous lesions because they provoke rearrangements in the genome and, consequently, gene conversion, cross-over and chromosome loss events. Some of these events can subsequently guide the cell to an aneuploid state, which is followed by mitotic arrest and leads to either secondary genomic changes as a result of aberrant cell division or cell death [1–3]. Diploid cells possess two copies of the genome and can tolerate changes that follow DNA breaks provided that all essential genes are present and the DNA imbalance does not preclude cell division. In haploid cells, the presence of a second copy of the genome that enables homologous recombination, which is the major DSB repair pathway in yeast, is only available when DNA is being replicated. Thus, the sensitivity to DSBs is greater in haploid cells than in diploid cells. The reported level of spontaneous gross chromosomal rearrangements (GCRs) observed in diploid cells is in the range of 10^-4^ to 10^-5^ per cell per generation [4,5], whereas in haploid cells, it is in the range of 10^-9^ to 10^-10^ per cell per generation [6–11]. Accordingly, GCRs occur 10^5^-fold more often in diploid than in haploid cells.

DNA strand breaks occur frequently in cells after γ-irradiation or chemical treatment with bleomycins, but they are also introduced into DNA intentionally under various physiological circumstances. During meiosis, 170 DSBs are initiated on average via enzymatic cleavage by Spo11, a topoisomerase II type enzyme [12,13]. These breaks are repaired via homologous recombination, which utilizes the sequence on the homologous chromosome as a template. Cross-over products, which often occur because of the repair process, are not only crucial for cell fertility but also promote homologous chromosome pairing, work together with sister chromatid cohesion to hold homologous chromosomes together until the onset of anaphase, and mediate the first meiotic chromosome segregation [14]. Meiotic crossovers create chiasmata, which are physical links between homologous chromosomes that facilitate their proper alignment at the metaphase plate and subsequent disjunction in anaphase of meiosis I [15].

A single DSB is also produced during yeast mating-type switching at the currently expressed mating-type locus, *MAT*. The homing endonuclease Ho is responsible for cleavage [16]. The repair of Ho-induced DSBs occurs via gene conversion using the chromosomally linked silent mating-type locus as a donor. SSBs are introduced regularly during various DNA repair pathways by a range of specialized endonucleases. For example, the Rad1-Rad10 complex and Rad2 are single-stranded DNA endonucleases that introduce SSBs during the repair of nucleotide excisions and DSBs [17–20]. Structure-selective endonucleases, such as Mus81, Yen1 or Slx4, cleave branched DNA and are involved in recombination and DNA repair [18,21–24]. The major apurinic/apyrimidinic endonuclease Apn1 functions in the repair of DNA damage caused by oxidation and alkylating agents [25]. Thus, the introduction of SSBs is a common natural process that occurs in various DNA damage repair pathways. SSBs can be easily converted into DSBs if they persist until the next round of replication [26–28]. SSBs are present in DNA at all times because of DNA damage or its repair and only differ in the type of free ends of the DNA molecules. SSBs and DSBs differ from each other depending on how they are generated. The greatest diversity of broken DNA ends appears after γ-irradiation or treatment with radiomimetic chemoenzymes [28]. Taking this observation into consideration, in our genomic screen for genes that are important for survival after DSB stress, we decided to use the radiomimetic compound zeocin.

Zeocin belongs to the bleomycins, which are a family of natural glycopeptides that are produced by *Streptomyces verticillus* and commonly used clinically as antitumor drugs. The therapeutic effectiveness of bleomycins is related to their ability to cleave nucleic acids, especially DNA, causing single-stranded and double-stranded DNA damage in the presence of required cofactors (metal ions, O_2_ and one-electron reducers). In a redox reaction introducing oxidative lesions into DNA, zeocin uses Cu^2+^ or Fe^3+^ ions as a cofactor [29,30]. It has been shown that DNA damage caused by bleomycins results in a variety of cleavage products. Nevertheless, the initial mixture of single-stranded and double-stranded nicks can be rapidly converted into DSBs; therefore, DSBs are the predominant type of lesions detected after treatment with these compounds.

The chemoenzymes belonging to the bleomycin group are not particularly specific, and they can introduce lesions in a broad range of macromolecules, perturb lipids and destroy proteins. It has also been shown that these chemoenzymes cleave selected members of all classes of RNA and subsequently impair protein synthesis [31]. Although bleomycin antibiotics are responsible for all of this cellular damage, their activity is still ascribed to their ability to bind DNA via intercalation with their planar bithiazole-containing moiety. DNA is then degraded by the metal ion-chelating portion of the molecule, which forms an active complex with metal ions and molecular oxygen [32,33].

Numerous cellular mechanisms have evolved to detect DNA lesions, minimize possible mutagenic changes and preserve genome integrity after the occurrence of damage. It is likely that separate genome maintenance mechanisms have evolved and been specifically adapted for haploid or diploid cells. It has been shown that the level of spontaneous mutagenesis in diploids is two orders of magnitude higher than in haploids [3,4]. A mutagenesis spectrum analysis conducted in haploid and diploid cells revealed profound differences between them; in haploids, most events leading to a loss of function of the *CAN1* or *URA3* marker gene are point mutations, such as base substitutions and frameshifts, whereas in diploids, most mutations are caused by recombination events, including gene conversions, cross-overs or chromosome losses. Because point mutations are present at the same level in diploid cells as in haploids (approximately 10^-6^, representing only 1% of all mutagenic events in diploids), rearrangements constitute the predominant class (99%) of all mutagenic events in these cells [4,5]. Because of this fact and the general belief that rearrangements occur from DSBs, it is expected that there are DNA repair mechanisms designed to detect and repair DSBs in diploid cells.

We performed a genome-wide screen using diploid strains from a yeast knock-out collection (YKO collection) [34], which were homozygous diploid for non-essential genes and heterozygous diploid for essential genes. To determine the deletion strains that display a decrease in survival after zeocin treatment, we employed barcode microarray technology [35,36]. The screen revealed 133 genes whose deletion sensitized the strains to zeocin. These genes are involved in a variety of biological processes, including stress response, DNA damage recognition, damage signal transduction, DNA repair pathways as well as pathways that coordinate DNA metabolism with the progression of the cell cycle, especially the control of cell division. Intriguingly, 49 genes involved in the vesicular trafficking system were also identified. Further analysis indicated that efficient vesicular trafficking is crucial for maintaining the genome because it contributes to protecting cells against zeocin accumulation and proper DSB recognition and repair, which prevents genome rearrangements. Our results predict multiple roles of vesicular trafficking in mechanisms involved in genome preservation under genotoxic stress conditions.

## Results and Discussion

### The search for genes whose deletion confers zeocin oversensitivity

Although the yeast *S*. *cerevisiae* is one of the best-explored models of eukaryotic cells in relation to genome maintenance mechanisms, considerable information on this topic remains to be determined, including the ploidy-specific response to genotoxic stress. As mentioned above, the two copies of the genome present in diploids make these cells hyper-recombinogenic in comparison with haploids, especially when genotoxic stress provokes the formation of DSBs. Although the body of knowledge on this topic is continually growing, our understanding of it is still far from complete. Consequently, it is expected that there are genes involved in the mechanisms responsible for protecting the cell against oxidative damage generated in DNA that have yet to be discovered. These mechanisms enable stress signal recognition and a cellular response that is adequate for the signal type and strength, which ensures that the genome is maintained in optimal form. In addition, numerous oxidative agents that cause severe DNA damage are frequently used in different anticancer therapies, and these therapies generally have harsh side effects. To shed more light on these issues, we studied the cellular commitments of zeocin, which is one of the oxidative agents, and applied a genome-wide approach using a *Saccharomyces cerevisiae* diploid yeast knock-out collection (YKO) generated by the *Saccharomyces* Genome Deletion Project Consortium [34] together with the barcode microarray technique. Our clone set included more than 5,000 homodiploid strains carrying double deletions of non-essential genes (HD) and approximately 1,100 heterodiploid strains in which one copy of an essential gene was removed from the cell genome (ESS). This approach is widely used to rank the oversensitivity or resistance of deletion clones to various agents on a genomic scale [37,38]. In the present study, we employed this method to screen the diploid YKO collection for clones showing zeocin oversensitivity. The diploid YKO strains were pooled to generate an equal number of cells of each deletion clone in the mixture. The clone pool that was grown to mid-exponential phase (1–2x10^7^ cells/ml) was exposed to one of two zeocin doses (5 or 15 μg/ml) for one hour. Under these conditions, the wild-type (WT) BY4743 strain showed 60% or 32% surviving clones on average. Interestingly, a 40- to 50-fold higher concentration of zeocin was required to achieve a similar decrease in the viability of post-diauxic cells. This difference is likely caused by a decrease in cellular permeability after the diauxic shift, which is the phenomenon that reduces the susceptibility of stationary cells to various compounds, which has been shown for H_2_O_2_ [39]. However, the possibility of exponentially growing cells (*i*.*e*., cells that are intensively replicating and transcribing DNA) being more sensitive to zeocin damage because of the higher susceptibility of their DNA to damage should also be considered. In our experiment, zeocin-treated cells and control (untreated) cells were harvested and washed, and 5x10^6^ cells were plated onto each of five 150 mm diameter synthetic complete medium (SC) plates. To ensure adequate representation of all deletion clones, the total number of cells in each biological replicate was at least 2.5x10^7^ (4,000 cells per deletion clone on average). The plates were incubated at 28°C for three days. We expected that oversensitive deletion clones would be represented by fewer cells than clones demonstrating a WT level of sensitivity to zeocin. Oversensitive clones were identified by comparing the relative representation of deletion clones in the pool treated with zeocin to that in the control pool with the barcode microarray technique. With this technique, the differences in strain representation were visualized through PCR amplification and fluorescent labeling of the barcodes of deletion strains from the zeocin-treated and control cells followed by a comparative hybridization of the resulting probes to barcode microarrays (see Materials and Methods for details). Oversensitive deletion clones were underrepresented in the zeocin-treated cell population compared with the control population, which could be identified by a lower level of fluorescence at the respective microarray spot. Four independent biological experiments were performed for every tested zeocin concentration, and there were two technical replicates with Cy3 and Cy5 dye swapping. The raw fluorescence data were normalized, and the average values for each zeocin concentration were calculated. For further analysis, 166 zeocin-oversensitive deletion strains that could be reproduced in a twofold or greater underrepresentation (compared with the control pool) in at least one zeocin-treated clone pool were selected (LogRatio<-1, p-value<0.1).

**Fig 1:**
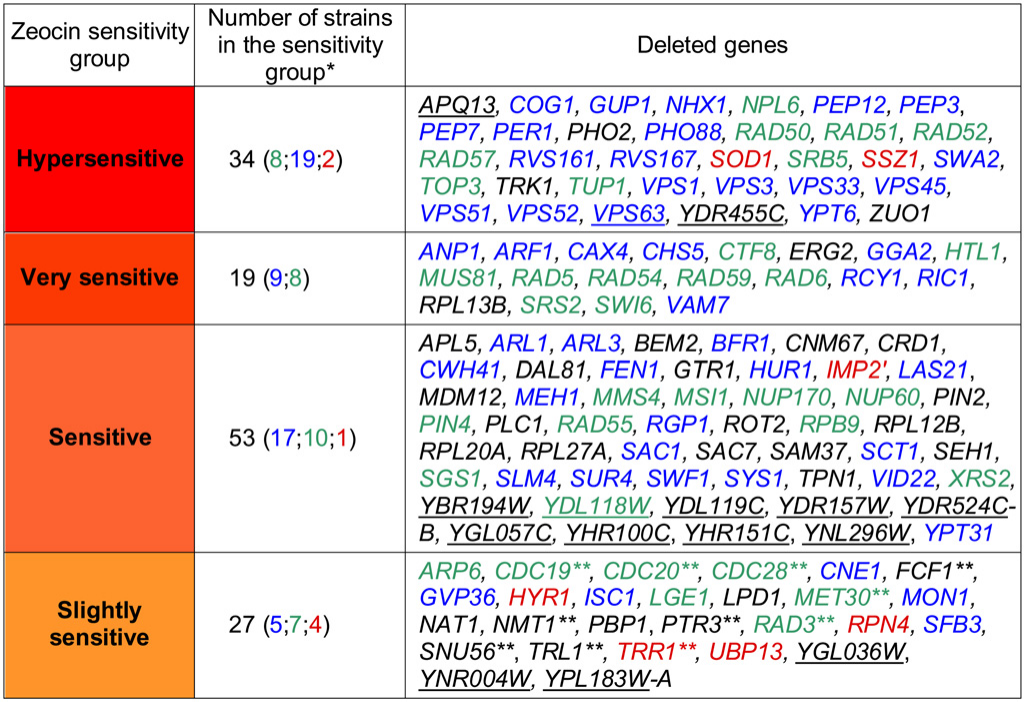
List of yeast deletion strains sensitive to zeocin. The zeocin-sensitive strains revealed in the genome-wide screen are divided into sensitivity groups according to their phenotype, which is assessed in the sensitivity test performed on individual diploid YKO strains. Genes with a documented role in genome maintenance are indicated in green; genes involved in vesicular trafficking are indicated in blue; genes involved in stress defense are indicated in red. ORFs of unknown function are underlined. * The number of genes belonging to each color-labeled category are given in brackets. ** Essential genes.

The inclusion in our screens of the diploid collection of clones lacking one copy of essential genes allowed us to distinguish potential gene dosage effects on zeocin oversensitivity and determine the lack-of-function phenotype that is detectable among the homozygous diploid (HD) clones. Our results indicated that 30 of the 166 (11 of 133 verified in individual tests) genes identified in the screen are essential (see Fig. 1).

### A semi-quantitative drop assay confirmed the zeocin oversensitivity of the majority of deletion clones identified in the screen

The 166 strains that were oversensitive to zeocin according to the microarray analysis were tested individually. Their survival on yeast extract peptone dextrose (YPD) medium supplemented with zeocin was analyzed in a semi-quantitative drop assay. Two concentrations of zeocin were used: 2.5 μg/ml and 5 μg/ml. The test confirmed the microarray results for 133 strains (approximately 80% of all strains identified in the screen). The strains showing survival below 80% on 2.5 μg/ml zeocin and below 70% on 5 μg/ml zeocin (i.e., strains exhibiting survival lower than 85% of the typical survival of the WT parental BY4743 strain in the same conditions) were classified as oversensitive. Moreover, individual analysis of the YKO strains permitted their classification with respect to the strength of the observed phenotype. We divided the YKO strains into four different sensitivity levels: (I) hypersensitive, (II) very sensitive, (III) sensitive and (IV) slightly sensitive. The 34 strains showing survival of approximately 0.1% of that observed in the WT were classified as hypersensitive, and 19 strains showing viability below 1% of that observed in the WT were classified as very sensitive. Moreover, the 53 strains showing viability below 15% of that observed in the WT were classified as sensitive (Fig. 1, S1 Fig.). The strains showing the slightly sensitive phenotype in the drop tests were subject to additional analysis, which finally verified their zeocin sensitivity phenotype. In the physiological experiment, oversensitivity to zeocin was confirmed for 27 strains (S1 Fig., S2 Fig.). Fig. 1 lists all of the genes (categorized by their sensitivity level) whose deletion caused decreased survival after zeocin treatment in individual tests.

### Computational analysis of the results of the zeocin oversensitivity screen revealed enrichment of two Gene Ontology categories: ‘genome maintenance’ and ‘vesicular trafficking’

All of the genes whose deletion resulted in a zeocin oversensitivity phenotype in individual tests were subjected to further analysis. Using Cytoscape with the BINGO plug-in [40], we performed analyses of the overrepresentation of Gene Ontology terms (S3 Fig. A-C). Several distinct functional terms were overrepresented, two of which dominated the biological process category (S3 Fig. A). The first corresponded to 33 genes involved in the DNA damage response, whereas the second corresponded to 49 genes involved in vesicular trafficking (see Fig. 1 for details). Analogous functional categories were found to be overrepresented in the analysis of the Gene Ontology terms molecular function and cellular compartment (S3 Fig. B and C, respectively). The results appeared to indicate that for survival under zeocin stress conditions, functional vesicular trafficking is as important as DNA damage recognition and repair pathways. This finding raises the question of the role of vesicular trafficking in protection against zeocin stress.

### DNA damage response genes involved in survival under zeocin stress

The genes that were identified as being important for the survival of zeocin stress in our screen (Fig. 1) would be expected to include genes whose products are involved in DNA damage signaling and repair. Indeed, the genes on our list included genes involved in DNA damage recognition, such as *RAD50* and *XRS2*, which are two subunits of the MRX complex and have been implicated in the processing of DSBs in vegetative cells as well as the initiation of meiotic DSBs and telomere maintenance. In *mrx* nuclease mutants, recombination is compromised because of the loss of MRX-associated processing and inhibitory effects of Yku70/Yku80 binding to the DSB ends [41]. Similar to γ-radiation enforced breaks, DSBs provoked by zeocin exhibit ‘ragged’ ‘dirty’ ends that require processing before the damage is repaired [42]. Such DSB ends are nonligatable; therefore, they cannot be the substrate for the NHEJ repair pathway [43], which may be one of the reasons that none of the genes involved in NHEJ appeared in the results of our genomic screen for zeocin oversensitivity. Similarly to haploid cells, the zeocin-generated ‘dirty’ DNA ends are readily resected in the G1 phase of the cell cycle because most DSBs in G1 cells do not have a repair template for homologous recombination (HR). Therefore, the non-clean ends in haploids are mostly repaired via HR in the S/G2 phase [43,44]. Moreover, it has been shown that in diploid cells, the efficiency of NHEJ is significantly reduced because of mating-type locus-dependent regulation of NHEJ activity [45,46], which might also explain the lack of representation of NHEJ in the results of our screen. Nevertheless, NHEJ is active in cell-cycle-arrested diploid cells and contributes to spontaneous mutagenesis [47]. The MRX complex has also been established as a co-factor for both the Mec1 and Tel1 DNA damage-dependent kinases, which are required for activation of the G1 and intra-S checkpoints after DSB formation [48]. Tel1 is able to trigger the G2/M checkpoint in an MRX-dependent manner in response to UV irradiation and in the absence of Mec1 [49].

Numerous genes involved in different pathways of DNA repair are included in our list. Notably, genes involved in all branches of homologous recombination (*RAD51*, *RAD52*, *RAD54*, *RAD55*, *RAD57*, *RAD59*) were strongly represented. The HR repair pathway is considered the most important pathway for the survival of yeast cells after the occurrence of DSBs, especially following zeocin treatment when the cells must address broken DNA ends that cannot be repaired via NHEJ. The appearance of HR genes in the list obtained through our zeocin oversensitivity screen authenticates our results. However, the screen also revealed other genes involved in various DNA repair pathways, some of which are involved in HR but in different steps of the repair pathway, such as in the resection of broken DNA ends or resolution of X structures. Additionally, our screen revealed genes encoding DNA helicases involved in recombination (*SRS2*, *SGS1*), replication fork regression during post-replication repair via template switching (*RAD5*) and nucleotide excision repair (*RAD3*). In addition, the screen revealed genes encoding both subunits of the structure-specific Mms4-Mus81 endonuclease, which cleaves branched DNA, and genes involved in recombination and DNA repair, such as topoisomerase III (*TOP3*); together with Sgs1, *TOP3* is involved in telomere stability and the regulation of mitotic recombination. Two genes whose products promote cohesion were also found: the *CTF8* gene encoding a subunit of the Replication Factor C-like complex, which is required for sister chromatid cohesion and enables DSB repair [50], and *HTL1* gene encoding a component of the chromatin structure remodeling (RSC) complex, which functions in the stability of chromosomes as well as in establishing sister chromatid cohesion and telomere maintenance [51,52]. In yeast, damage-induced cohesion is essential for repair in postreplicative cells. Moreover, genome-wide cohesion is established after the induction of a single DSB and controlled by the DNA damage checkpoint and cohesin-regulating factors independent of DNA duplication. This cohesion acts together with the cohesion generated during replication in sister chromatid—based DSB repair [53]. Thus, the role of cohesion in DSB repair, particularly in the proper timing of this event when it occurs outside of mitosis, was underpinned by the results of our screen.

Our zeocin oversensitivity screen also revealed a group of factors that are responsible for cellular homeostasis and involved in the control of the cell cycle or cell division, including *CDC28*, *CDC20* and *SWI6*. Cdc28 is a catalytic subunit of the main cell-cycle cyclin-dependent protein kinase (CDK) that alternately associates with different cyclins (Cln and Clb), which direct CDK to its specific substrates (e.g., Cdc20 and Swi6). CDK is essential for traversing the START checkpoint, which is when cells enter the cell cycle from the G1 state, and providing G2/M checkpoint control [54,55]. It has been shown that a *cdc28* mutant arrested in G1 is significantly more sensitive to hydrogen peroxide than other *cdc* mutants arrested in later phases, including G2 [56]. Published results reveal variable cellular responses to different stresses, such as UV irradiation, ionizing radiation, hydrogen peroxide, and menadione, all of which cause DNA damage through the formation of hydroxyl radicals. This variability suggests that the components of the cell-cycle arrest mechanisms related to such stress responses are extremely specific. In addition, because all of these stresses cause similar types of DNA damage, it remains unclear if the cell-cycle arrest in response to these stresses results from DNA damage or another trigger [56]. Another protein that is involved in cell-cycle progression and required for M/G1 timing as well as for START is Swi6, which can sense various DNA damage events and react to them differently [54]. Swi6 has been shown to regulate cell-cycle arrest upon DNA damage following phosphorylation by Rad53 [57]. However, the mechanisms underlying the function of Swi6 in regulating cell-cycle arrest in response to oxidative stress are diverse and independent of the DNA damage and repair pathway [56]. It has been shown that Swi6 can sense oxidative stress through an intrinsically reactive cysteine residue and regulate the cell cycle by delaying the exit from G1; thus, cellular damage can be removed or repaired to prevent the damage from being passed on to daughter cells [58]. Another Cdc28 substrate that emerged from the screen is Cdc20, which is a cell-cycle-regulated activator of the anaphase-promoting complex required for the direct ubiquitination of mitotic cyclins (a homologue of β-transducin). Similarly to Pds1 and other anaphase inhibitors, the Cdc20 protein is responsible for controlling the G2/M checkpoint that prevents mitosis in response to DNA damage by inhibiting nuclear division. [59]. The results of our screen confirm that cell-cycle initiation and progression are tightly controlled to guarantee that cell division does not occur under risky conditions, such as oxidative stress, and highlight the factors responsible for linking oxidative stress to the cell cycle.

The screen also revealed that the *SOD1* and *TRR1* genes that encode superoxide dismutase and thioredoxin reductase, respectively, contribute to the cellular stress response by conferring enzymatic protection against oxidative damage. Genes that are involved in the clean-up process after stress effects, such as *CNM67* and *ZUO1*, which encode chaperones, and *RPN4*, which encodes a transcription factor that up-regulates the expression of 26S proteasome genes in response to various stresses and stimulates the degradation of damaged proteins, were also identified. Interestingly, Rpn4, which regulates proteasomal gene expression, is also a proteasomally degraded protein. The disruption of either of the two branches of the Rpn4-proteasome negative feedback loop, including Rpn4-induced proteasome expression or proteasomal degradation of Rpn4, severely impairs cell viability under stressful conditions [60]. Higher processivity of the 26S proteasome is required after an increase in the damage to cellular proteins, such as after oxidative stress. Zeocin entering into the cell produces a toxic effect, which requires efficient removal of zeocin-dependent damage. However, Rpn4 has also been shown to be directly involved in nonhomologous end-joining (NHEJ). Rpn4, which is a DNA-binding protein, is directly recruited to DSBs, and because of its capacity to bind the proteasome, it can target the proteasome to the damage site, thus influencing NHEJ [60].

### Vesicular trafficking is potentially involved in genome maintenance

Another large group of genes that were found to be important for survival after zeocin treatment consisted of genes involved in vesicular trafficking. Almost all branches of vesicular trafficking were represented in this group of genes except for components of the endosomal sorting complex required for transport (ESCRT) (S3 Fig. C). These genes can be divided into three thematic groups: genes related to autophagy, genes associated with the organization of the actin cytoskeleton and genes related to cellular pH homeostasis. In each subgroup, genes encoding potential Cdc28 substrates were found (*MON1*, *SAC7* and *SFB3 (LST1)*), suggesting that all three processes must be regulated during the cell cycle [61].

*ARF* genes, which are the canonical genes linked to this process, were not found among the genes involved in autophagy, but a range of genes were identified whose products have been shown to contribute to this process, such as the *MON1* gene, which encodes a protein required for the fusion of cytoplasm-to-vacuole-targeting Cvt-vesicles and autophagosomes with the vacuole, and *MEH1* and *SLM4*, which are two components of the EGO complex involved in the regulation of microautophagy. Other genes such as *HUR1*, *PEP12*, *VAM7*, *VPS51* and *VPS52* were also previously described as being involved in autophagy [62,63], and some of their gene products are involved in vesicle formation, flow or fusion; therefore, they may be implicated in other pathways of vesicular trafficking. The inclusion of autophagy-connected genes in the results of our screen may be explained by the fact that oxidative stress fills the cell with damaged proteins that must be removed quickly or they will cause secondary injury to the cell. Thus, all of the autophagy-related gene products mentioned above would function in parallel with the 26S proteasome. Alternatively, these results may be explained by implicated roles of autophagy in the regulation of the cell cycle, DNA repair pathways and genome maintenance, which is similar to the 26S proteasome. The proteasome may act selectively through specific receptors, such as Rad23 and Rpn10, by recognizing certain types of ubiquitin conjugates [64] or selecting specific proteins for degradation at the indicated time point during the cell cycle or under a particular stress. This type of selective degradation has been demonstrated to occur for Eco1, Ho nuclease, DNA polymerase Rad30, and stress-related transcription factor Rpn4 [65–68]. Several lines of evidence suggest similar capabilities of autophagy. Mutations in the ESCRT complex components *vps25* and *vps36* have been shown to increase topoisomerase I levels, which are dependent on small ubiquitin-like modifier (SUMO) modification [69]. In addition, the inhibition of histone deacetylase has been shown to specifically counteract yeast Mec1 activation, DSB processing and ssDNA—RPA nucleofilament formation. Moreover, the recombination protein Sae2 is acetylated and degraded following histone deacetylase inhibition. Therefore, histone modification controls chromosome stability by coordinating the Mec1-dependent checkpoint and DSB processing through autophagy [70]. In our zeocin oversensitivity screen, components of the ESCRT complex, histone acetyltransferases and deacetylases were not detected; nevertheless, the data presented above constitute one of the few well-documented examples of the influence of vesicular transport on the stability of the yeast cell genome [69,70]. Considering that every damage response is unique and tailored to the damaging insult, we also expect a specialized vesicle-mediated response to zeocin treatment.

Zeocin acts as a radiomimetic and causes lesions that are preferentially recognized and repaired by the HR machinery, which is similar to the results of irradiation [43]. Accordingly, our screen identified almost all of the genes involved in HR. In addition, published data indicate that the formation of recombination foci at zeocin-induced DSBs requires an exit from G1 and neither the initiation of DNA replication nor the presence of homologous sequences is necessary [71]. As mentioned above, the results of our screen include several factors guiding the G1/S transition. In addition, genes implicated in a special autophagy pathway, piecemeal microautophagy of the nucleus (PMN), were identified [62,72], suggesting that this process is involved in the response to DNA damage. This phenomenon would resemble micronucleus formation and degradation via the mechanism referred to as programmed DNA degradation, which is observed in mammalian cells after severe DNA damage, especially after DSB formation [73–75]. The screen also revealed genes involved in cytoplasm-to-vacuole-targeting (Cvt; *VPS51*, *VPS52*, *VPS45*), homotypic vacuole fusion (*VAM7* and *YPT7*) and sphingolipid biosynthesis (*FEN1*, *SUR4*).

Among the genes associated with the actin cytoskeleton that were indicated in the zeocin oversensitivity screen, we identified *SAC7*, which encodes a protein that activates Rho1 GTPase, a component involved in signaling to the actin cytoskeleton; *VPS1*, which encodes a dynamin-like GTPase, a component involved in organizing the actin cytoskeleton; and several genes required for polarization of the actin cytoskeleton, including *GVP36*, *RVS161*, *RVS167*, *VPS51* and *VPS52*; the latter two genes encode components of the Golgi-associated retrograde protein (GARP) complex, which is involved in actin localization [76]. The actin cytoskeleton is necessary for numerous intracellular processes, and it is difficult to determine the relevant processes under zeocin treatment. The most obvious link would appear to be endocytosis, but the link could also be the movement of vesicles carrying protein and membrane cargo within the cell.

The following gene products that are associated with cellular pH were identified in our zeocin oversensitivity screen: Sfb3, which together with Sec23 is involved in the sorting of Pma1 into COPII vesicles; and Gga2, which together with Gga1 functions as an adaptor required for the recognition of Pma1 via ubiquitin-dependent and ubiquitin-independent binding and missorting of Pma1 in response to changes in nutrition [77]. Pma1 is the plasma membrane H^+^-ATPase that pumps protons out of the cell. The Pma1 protein is a major regulator of cytoplasmic pH and the plasma membrane potential, and it is also a part of the P2 subgroup of cation-transporting ATPases [78]. In addition to Pma1, Gga2 also functions as an adaptor for ferrichrome transporter Arn1. In the absence of ferrichrome, Arn1 should be sorted directly from the *trans*-Golgi network to the vacuolar lumen via the vacuolar protein-sorting pathway; however, in *gga2* cells, it is missorted to the plasma membrane, which causes a loss of control in iron homeostasis and provokes oxidative stress in the cell [79]. pH has been shown to regulate the functioning of a variety of transporters and permeases as well as protein trafficking, and it also aids in metal ion homeostasis and supports cellular mechanisms of resistance to a multitude of chemical stresses.

### Changes in the electrochemical gradient can moderate sensitivity to zeocin in certain deletion strains

It is believed that any mutation resulting in an alteration of the electrochemical gradient leads to anomalous sensitivity to all cationic drugs independent of their mechanism of toxicity [80]. Over half of the mutant strains that display an altered tolerance to hygromycin B, spermine, and tetramethylammonium present strong or moderate growth defects at a limiting K^+^ concentration. Zeocin is a drug containing the metal ion cofactor Cu^2+^; therefore, cells may have a reaction to zeocin that resembles their reaction to the previously described cationic drugs. However, the data obtained in a genome-wide screen for bleomycin-resistant mutations showed that bleomycin uptake depends on the L-carnitine transporter Agp2 rather than on proteins, which ensures a proper electrochemical gradient [81]. A similar mechanism of action might be expected for zeocin, which is a chemical from the same group. However, in our zeocin oversensitivity screen, we did not observe overrepresentation of the *agp2*Δ strain. The uptake of zeocin likely depends on the same protein, but it was impossible to select *AGP2* in our screen because the respective deletion strain is missing from the homozygous diploid knock out collection that we used in our study. Moreover, our results do not indicate a requirement for zeocin uptake of any other specific transporter protein. However, mouse cells (HEK293) that are normally sensitive to zeocin become completely resistant to this compound in the presence of an elevated K^+^ ion concentration, which depolarizes the resting membrane potential [82]. This finding suggests the involvement of an electrochemical gradient in the zeocin uptake process, which is consistent with the participation of an ion channel, transporter, or exchanger in mammalian cells. Our own data also implicate the involvement of an electrochemical gradient in zeocin sensitivity/resistance (see the discussion on Sfb3, Sec23, Gga2 and Gga1 above); therefore, we have concluded that the influence of K^+^ ions on zeocin sensitivity requires further study.

**Fig 2:**
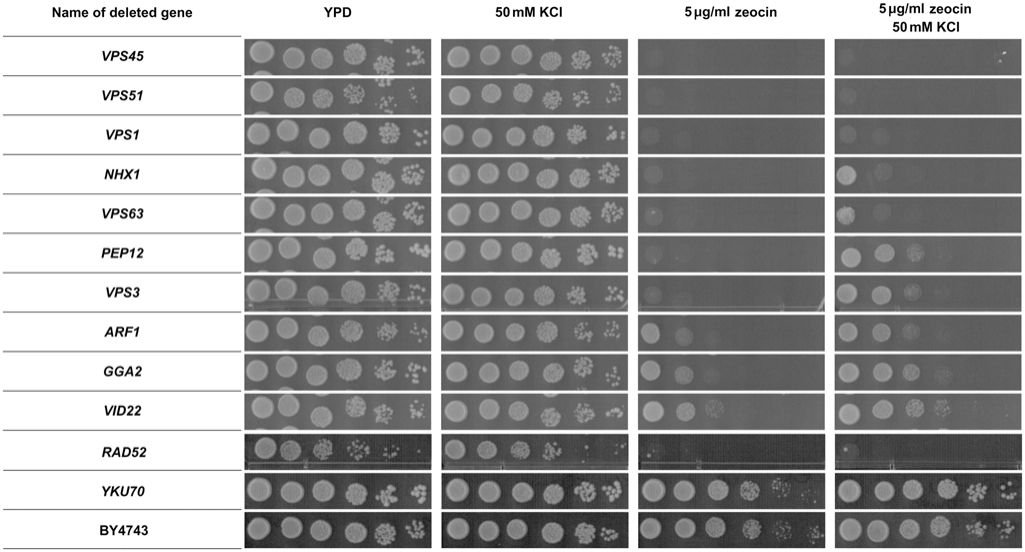
Results of the zeocin sensitivity and KCl suppression assays for *de novo*-constructed deletion strains. The strains containing deletions of the *VPS45*, *VPS51*, *VPS1*, *NHX1*, *VPS63*, *PEP12*, *VPS3*, *ARF1*, *GGA2* and *VID22* vesicular trafficking-related genes in a 2n background. Cell suspensions were serially diluted and spotted onto selection plates with either 5 μg/ml zeocin, 50 mM KCl or both compounds, and they were also spotted onto dilution control plates as described in the Materials and Methods.

To resolve this problem, we tested all 133 strains for zeocin sensitivity in the presence of 50 mM KCl. The elevated extracellular level of K^+^ ions should help zeocin-oversensitive strains grow during treatment provided that the gene deletions they carry have no impact on zeocin turnover. Strains lacking genes involved in zeocin detoxification or genes that are responsible for the repair of drug-mediated damage might not be suppressed by KCl during zeocin treatment. The results showed diverse sensitivity patterns among the tested deletion mutants (see Table 1, S1 Fig. and Fig. 2). All of the possible phenotypes were observed in the 133 deletion strains, including full suppression of zeocin oversensitivity by K^+^ ions (63), various degrees of partial suppression (51), and no suppression whatsoever (19). The full-suppression phenotype was observed frequently in the strains showing lower sensitivity to zeocin, whereas a lack of suppression was more common in the highly sensitive strains. Interestingly, no correlation was observed between the sensitivity pattern of the deletion strain and function of the deleted gene. The strains bearing mutations in genes involved in DNA maintenance and vesicular trafficking displayed all possible levels of KCl suppression. A good example is provided by the strains lacking functions required for a proper DNA damage response. The *XRS2-*, *MMS4-* and *SGS1*-deleted strains were moderately sensitive to zeocin and fully suppressed by KCl, whereas the *RAD50-*, *RAD51-*, and *TOP3*-deleted strains exhibited high sensitivity to zeocin treatment and none to very slight KCl suppression. However, the *RAD6-*, *RAD59-* and *SRS2*-deleted strains, which were also sensitive to zeocin, were suppressed by KCl.

**Table 1:** Various levels of suppression of zeocin sensitivity by KCl are detected in yeast deletion strains.

Zeocin sensitivity group	KCl effect	Deleted genes
Hypersensitive	No suppression	***PEP3***, *PHO2*, *RAD50*, *RAD51*, *RAD52*, *RAD57*, ***RVS167***, *SSZ1*, ***SWA2***, *TUP1*, ***VPS1***, ***VPS33***, ***VPS45***, ***VPS51***, *ZUO1*
	Low suppression	*APQ13*, ***RVS161***, *SRB5*, *TOP3*
	Partial suppression	***COG1***, ***GUP1***, ***NHX1***, *NPL6*, ***PEP12***, ***PEP7***, ***PER1***, ***PHO88***, *SOD1*, ***VPS52***, ***VPS63***, *YDR455C*, ***YPT6***
	High suppression	*TRK1*, ***VPS3***
Very sensitive	No suppression	*RAD54*, *RPL13B*
	Partial suppression	***ANP1***, ***ARF1***, *CTF8*, *ERG2*, ***GGA2***, *HTL1*, *RAD5*, *RAD59*, *RAD6*, ***RIC1***, *SRS2*, *SWI6*
	High suppression	***CAX4***, ***CHS5***, *MUS81*, ***RCY1***, ***VAM7***
Sensitive	No suppression	*IMP2'*, ***SUR4***
	Low suppression	*MDM12*, *RPB9*, *YDL118W*
	Partial suppression	*APL5*, *DAL81*, *PLC1*, *RPL27A*, *TPN1*, ***VID22***
	High suppression	*CNM67*, ***FEN1***, ***RGP1***, ***SAC1***
	Full suppression	***ARL1***, ***ARL3***, *BEM2*, ***BFR1***, *CRD1*, ***CWH41***, *GTR1*, ***HUR1***, ***LAS21***, ***MEH1***, *MMS4*, *MSI1*, *NUP170*, *NUP60*, *PIN2*, *PIN4*, *RAD55*, *ROT2*, *RPL12B*, *RPL20A*, *SAC7*, *SAM37*, ***SCT1***, *SEH1*, *SGS1*, ***SLM4***, ***SWF1***, ***SYS1***, *XRS2*, *YBR194W*, *YDL119C*, *YDR157W*, *YDR524C-B*, *YGL057C*, *YHR151C*, *YNL296W*, ***YPT31***, *YHR100C*
Slightly sensitive	Partial suppression	*LPD1*, *HYR1*
	Full suppression	*ARP6*, *CDC19*, *CDC20*, *CDC28*, ***CNE1***, *FCF1*, ***GVP36***, ***ISC1***, *LGE1*, *MET30*, ***MON1***, *NAT1*, *NMT1*, *PBP1*, *PTR3*, *RAD3*, *RPN4*, ***SFB3***, *SNU56*, *TRL1*, *TRR1*, *UBP13*, *YGL036W*, *YNR004W*, *YPL183W-A*

The zeocin-sensitive strains revealed in the genome-wide screen are divided into groups according to their ability to suppress the zeocin sensitivity phenotype on medium containing 50 mM KCl. The genes encoding products involved in vesicular trafficking are indicated with bold font.

This example clearly demonstrates a condition-dependent adjustment of the cellular response because enzymes that have a similar enzymatic activity (e.g., DNA helicases Srs2 and Sgs1) or are components of the same repair pathway (e.g., HR proteins Rad52, Rad59) or same functional complex (e.g., Rad50 and Xrs2) may cause different phenotypes when deleted. This finding could be explained by the highly specialized division of cellular pathways in response to a particular stimulus and by the diversity of roles these proteins may play in many aspects of DNA metabolism. However, it is still impressive how various proteins or parts of different pathways respond with precise activity to a particular stimulus and how this response fits the best actual conditions to preserve cellular integrity. Consequently, we observe the remarkable phenomenon of homeostasis.

The results of the KCl suppression assay revealed an absolute requirement for the HR genes *RAD51*, *RAD52*, *RAD54* and *RAD57* for zeocin stress survival (see the ‘no suppression’ category in Table 1). The results also predict a special role in protection against zeocin for *PHO2*, *TUP1*, *ZUO1*, *RPL13B*, *IMP2’*, *PEP3*, *RVS167*, *VPS1*, *VPS33*, *VPS45*, *VPS51* and *SUR4* (‘no suppression’ category) and suggest a similar requirement for *TOP3*, *APQ13*, *SRB5*, *MDM12*, *RBP9*, *YDL118W* and *RVS161* (‘low suppression’ category). Although certain genes listed above are required for survival after zeocin treatment, such as the genes involved in HR, because they play an established role in DSB repair, the requirement for genes involved in vesicular trafficking to resist zeocin stress is rather surprising. The results of the KCl suppression assay suggest two possible sources of zeocin oversensitivity: (1) an elevated intracellular level of zeocin caused by excessive uptake, decreased removal or impaired detoxification; and (2) improper recognition, signaling or repair of zeocin-dependent damage. Our data also indicate that at least two different mechanisms of zeocin oversensitivity occur: one involves the membrane potential and the other is membrane-potential independent. Additionally, our results indicate that vesicular trafficking is involved to some extent in protection against zeocin stress and suggest that it may play a direct role in genome maintenance processes.

### Increased zeocin sensitivity of strains with impaired vesicular trafficking cannot be explained by an elevated level of intracellular zeocin

To obtain additional insight into the mechanisms by which impaired vesicular trafficking influences zeocin oversensitivity, we decided to estimate intracellular zeocin levels in various deletion mutants.

**Fig 3:**
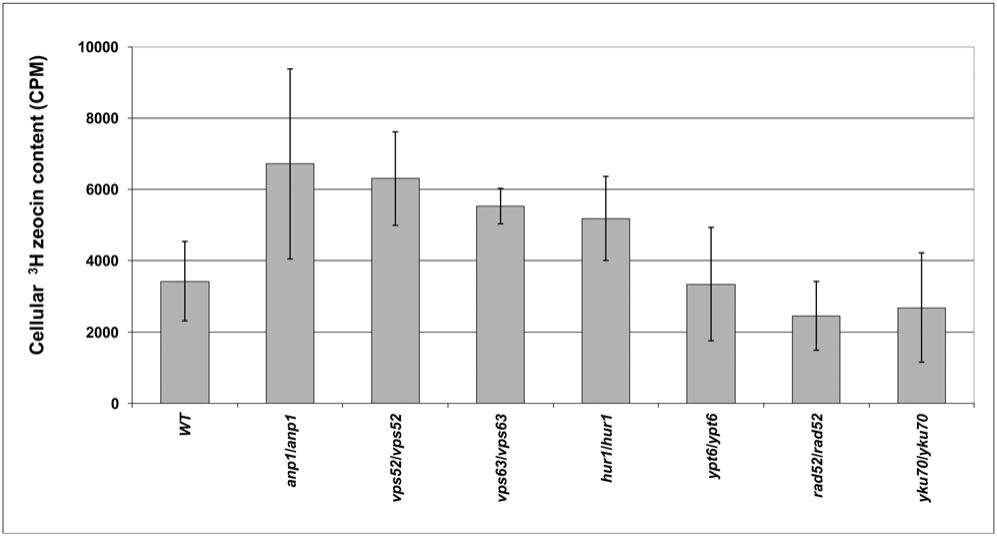
Intracellular levels of ^3^H-zeocin. Diploid strains lacking *ANP1*, *VPS52*, *HUR1* and *YKU70* from the diploid YKO collection in the BY4743 background and their newly prepared equivalents in a 2n background. YAS330 (*rad52*:*HIS3/rad52*:*HIS3*) and YMS20 (*vps63/vps63*) were treated with 15 μg per ml of ^3^H-zeocin for one hour followed by measurement of the intracellular levels of ^3^H-zeocin (see the Materials and Methods for details). The BY4743 (WT), *rad52*::*HIS3/rad52*::*HIS3* and *yku70*/*yku70* strains served as controls. The histogram shows the average and standard deviation calculated from the CPM measurements from at least four independent samples for each strain.

The intracellular levels of zeocin were determined for all of the deletion strains lacking vesicular trafficking genes selected in our screen and compared with the WT strain and two additional control strains lacking genes involved in DNA repair, with one displaying zeocin oversensitivity and the other presenting a WT response to zeocin (*rad52/rad52* and *yku70/yku70*, respectively). For this test, we employed radiolabeled ^3^H-zeocin (see Materials and Methods for details). Almost all of the tested strains, including both the *rad52/rad52* and *yku70/yku70* control strains, displayed a cellular H^3^-zeocin content similar to the WT levels (S4 Fig. B). Only four of the strains that lacked the *ANP1*, *VPS52*, *VPS63* and *HUR1* genes exhibited an increase in the intracellular level of H^3^-zeocin (Fig. 3). Two of these genes contribute to cell-wall biogenesis. Vps52 is involved in the localization of chitin [83], and Anp1 displays alpha-1,6 mannosyltransferase activity [84]. The other two strains that accumulated zeocin harbored deletions of open reading frames (ORFs) of unknown function that overlap well-known genes. *VPS63* almost fully (98%) overlapped the *YPT6* gene. The deletion of *VPS63* effectively removed the C-terminal half of *YPT6*. Interestingly, the complete deletion of *YPT6* also increased zeocin sensitivity, but the *ypt6/ypt6* deletion strain did not accumulate zeocin (see Fig. 3). Approximately half of *HUR1* overlapped the C-terminus of the *PMR1* gene, and its deletion resulted in the synthesis of a truncated Pmr1 protein. Ypt6 and Pmr1 are involved in protein sorting and exocytosis [85,86]. All four strains that accumulated zeocin displayed increased sensitivity to numerous chemicals [87], which suggests that the affected genes are required for general resistance to chemical insult.

Because the great majority of the analyzed strains showed no difference or even a decrease in the intracellular zeocin level compared with the WT cells, we can reasonably conclude that the oversensitivity of the tested strains to this toxic compound cannot be ascribed to either excessive uptake or decreased removal of zeocin from the cell. Therefore, this oversensitivity must be caused by the involvement of vesicular trafficking-associated genes in cell protection against zeocin-initiated damage.

Considering the results of the radiolabeled H^3^-zeocin experiment and KCl suppression assay together, we can conclude that the anticipated impact of varying zeocin uptake among individual deletion strains was negligible. In most cases, the oversensitivity of the analyzed deletion strains to zeocin did not coincide with changes in zeocin uptake.

### Elevated ROS levels cannot explain the zeocin oversensitivity of the majority of vesicular trafficking-impaired strains

**Fig 4:**
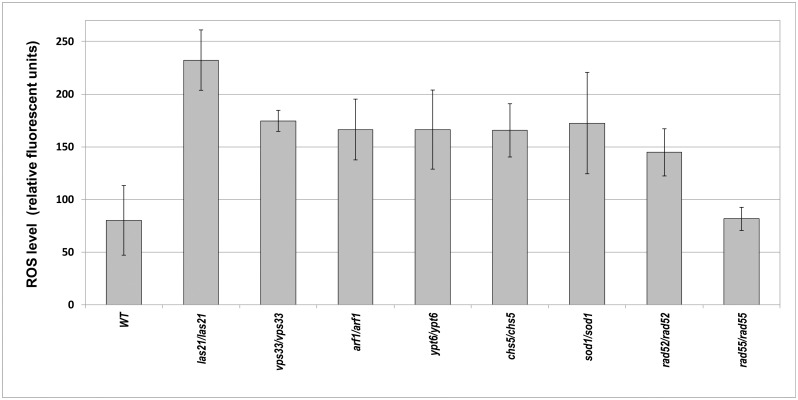
The ROS levels in analyzed diploid deletion strains. Intracellular endogenous ROS levels in exponentially grown diploid strains lacking *LAS21*, *VPS33*, *YPT6*, *CHS5*, *SOD1*, and *RAD55* in the BY4743 background and their newly prepared equivalents in a 2n background. YAS330 (*rad52*::*HIS3/rad52*::*HIS3*) and YMS16 (*arf1/arf1*). BY4743 (WT) and the *sod1/sod1* strain served as controls for the WT and elevated ROS levels, respectively. The data for the *rad52*::*HIS3/rad52*::*HIS3* and *rad55/rad55* strains defective in HR are shown for comparison. Intracellular ROS levels were determined fluorometrically with DCFH-DA as described in the Materials and Methods. Relative fluorescence units were normalized to the number of cells used in the assay. The histogram shows the average of at least four independent measurements for each strain, and error bars represent the standard deviation.

The toxicity of zeocin can be augmented if the strain subjected to treatment shows increased levels of endogenous reactive oxygen species (ROS). In such cases, the observed decrease in cell survival is caused by the additive effect of zeocin treatment and impaired redox homeostasis in the particular deletion strain. To verify this hypothesis, we measured the endogenous ROS levels in all of the zeocin-oversensitive strains lacking various vesicular trafficking genes through a fluorometric assay and employed 2,7-dichloro-dihydro-fluorescein diacetate (DCFH-DA) as a fluorescent ROS indicator. These tests led to the intriguing finding that in most cases, impaired vesicular transport diminished cellular ROS levels (S4 Fig. C). Increased ROS levels were detected in only a handful of strains with deletions of the following genes: *LAS21*, *VPS33*, *ARF1*, *YPT6* and *CHS5* (see Fig. 4). All of these strains showed an ROS level similar to that observed in the strain lacking *SOD1*, which encodes a cytosolic copper-zinc superoxide dismutase, one of the most important enzymes for cellular defense against oxidative stress because it detoxifies superoxide ions [88]. The elevation of endogenous ROS might be expected in the *arf1/arf1* strain, in which iron homeostasis is affected because of high uptake of this element via the siderophore pathway and reductive pathway [89]. Thus, the cellular ROS level can increase because of excess iron. Three other mutant strains, *csh5/csh5*, *vps33/vps33* and *ypt6/ypt6*, exhibited increased glutathione excretion [90]. Glutathione acts as a redox buffer, and its reduced form competes for the oxidizing equivalents derived from the disulfide bond-forming machinery, thereby minimizing the generation of cellular ROS [91].

The control DNA repair-deficient strain, *rad52Δ/rad52Δ*, which lacks the Rad52 protein that is involved in HR, also showed a significant twofold increase in its endogenous ROS levels compared with the WT strain. This result sheds new light on previously published data concerning the response of strains lacking Rad52 to ROS-generating conditions. The hypersensitivity of the *rad52/rad52* strain to free radicals [92] and co-lethality of the absence of Rad52 and Tsa1, a key cellular peroxiredoxin [93], might be explained by the synergistic deleterious effects of increased ROS levels in *rad52Δ* and decreased repair ability of this strain. This interpretation is supported by the fact that the *rad55/rad55* strain, which lacks another protein involved in HR, displays ROS levels similar to the WT strain. Thus, the high level of endogenous ROS observed in *rad52/rad52* cells appears to be specific to this deficiency (see Fig. 4). The cause of this increase is unknown.

### Strains with impaired vesicular trafficking display a genome instability phenotype

**Fig 5:**
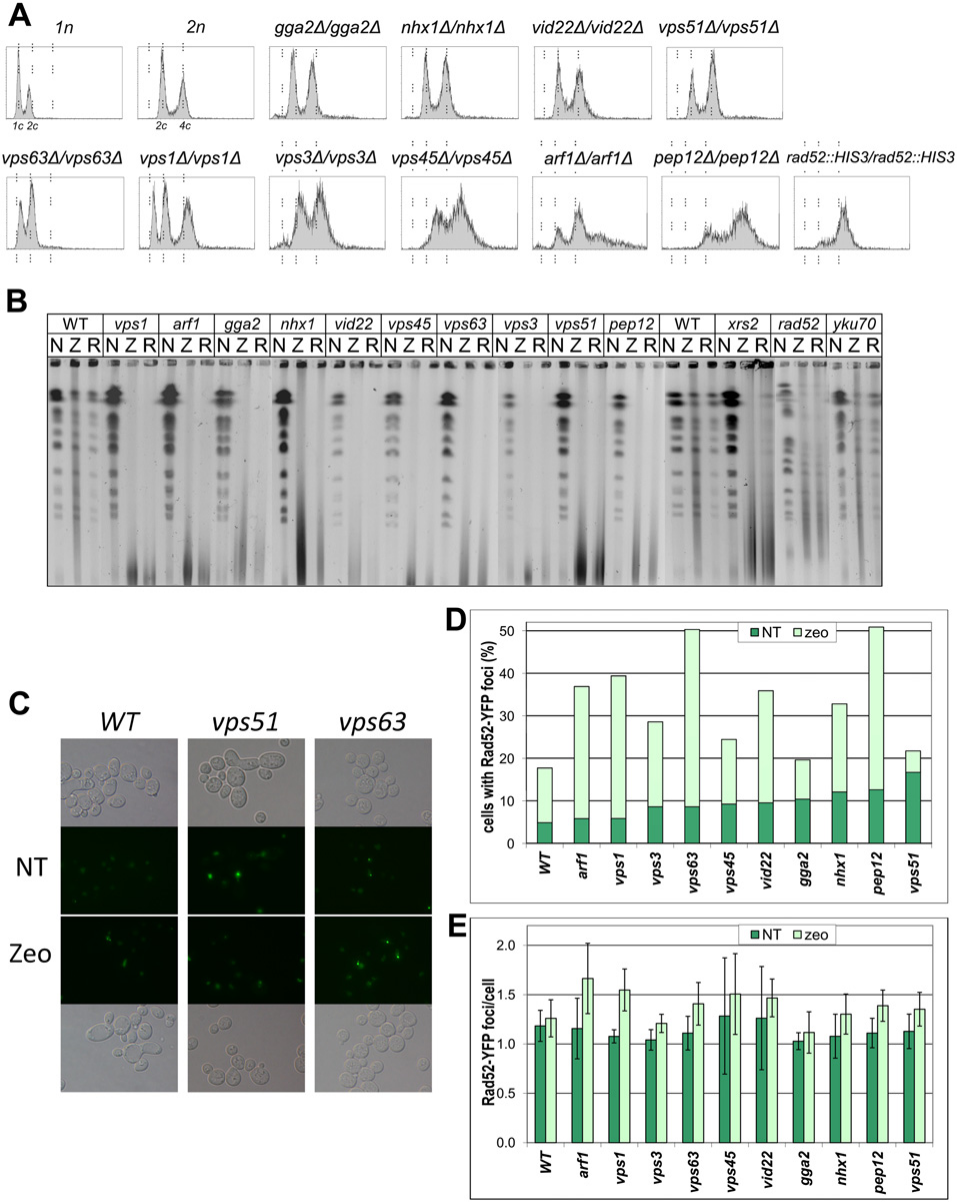
Genome instability phenotypes of zeocin-sensitive diploid strains lacking vesicular trafficking genes. The analysis was performed with *de novo*-constructed strains in a 2n background. (A) Results of the DNA content analysis via FACS. Yeast cells were stained with propidium iodide and and Methods. WT 1n and 2n strains served as DNA content controls. (B) PFGE analysis of yeast chromosome integrity after zeocin treatment. Exponentially growing cells were synchronized for 3 hours with 15 μg per ml nocodazole (N) or treated with 150 μg per ml zeocin for one hour (Z), and after the removal of zeocin, they were allowed to recover for 3 additional hours of growth in medium supplemented with 15 μg per ml nocodazole (R). The 1n (WT) strain served as a positive control strain, and the *rad52/rad52* and *xrs2/xrs2* strains served as negative controls that were unable to recover from zeocin stress. *yku70/yku70* was included in the analysis as a zeocin-insensitive strain. (C-E) Induction of nuclear Rad52-YFP foci by zeocin in the haploid strains with deleted vesicular trafficking genes in the BY4741 background. (C) Microscopic images of cells expressing the Rad52-YFP fusion protein from the pWJ1344 plasmid visualized after one hour of treatment with 150 μg/ml zeocin. The formation of Rad52-YFP foci in WT (left column) and two selected strains: *vps51*, showing an elevated level of spontaneous formation of Rad52-YFP foci (middle column), and *vps63*, displaying an increase in the number of cells with Rad52-YFP foci. Bright-field images displaying the distribution of cells (upper row) and fluorescent pseudocolored monochrome images with Rad52-YFP in green (lower row) are shown. (D-E) Quantification of Rad52 foci. All focal planes were inspected for Rad52 foci in at least 600 cells per analyzed strain. The number of foci observed before and after zeocin treatment was counted, and the percentage of cells with at least one Rad52-YFP focus (D) and average number of foci per cell (E) were calculated.

DSBs are the most serious type of zeocin-induced damage in cells and may provoke gross chromosomal rearrangements (GCR); therefore, we decided to assess the incidence of these events in zeocin-oversensitive strains through a DNA content analysis performed via flow cytometry. We conducted a fluorescence-activated cell sorting (FACS) analysis following propidium iodide staining of genomic DNA to check for possible DNA content aberrations in the cells of zeocin-oversensitive strains harboring deletions of genes involved in vesicular trafficking. Among the 49 diploids lacking genes implicated in various aspects of vesicular transport, approximately half (25 strains) displayed DNA content abnormalities (S4 Fig. A). Interestingly, the FACS analysis of these strains also revealed varying trends in the observed DNA content changes. Certain strains manifested a tendency to lose DNA and presented a DNA content histogram that is typical of haploids (e.g., *vps1/vps1*, *vid22/vid22*, *cog1/cog1* and *pho88/pho88*). A similar ploidy reduction was previously reported for several diploid *S*. *cerevisiae* deletion strains, including *ctf18/ctf18* and *rad52/rad52*, but the same final effect is produced by a different mechanism in each of these cases. In the *ctf18/ctf18* strain, genome reduction appears to occur in a single step and is most likely a result of selection pressure, with ploidy reduction employed as an escape mechanism in case of rearrangement stress [3]. In the *rad52/rad52* strain, ploidy reduction is achieved in sequential steps through the loss of chromosomes one at a time [94]. However, it is unclear which of these two mechanisms is employed in the haploidization of initially diploid cells harboring deletions of the *VPS1*, *VPS51*, *COG1*, *PHO88*, *HUR1* and *MEH1* genes. Although the strains mentioned above displayed a DNA content typical of haploids, sub-G1 peak growth within the diploid population was clearly visible in the histograms obtained for cells lacking the *LAS21* gene and, as a small population, for cells lacking the *GGA2*, *RVS161* and *PER1* genes. This sub-G1 population also appeared in the DNA content histograms for the *de novo*-prepared *gga2/gga2* strain, emphasizing the significance of this cell fraction (data not shown). A similar sub-G1 peak build-up within the diploid population could be observed in the FACS profile of the freshly prepared homozygous *vps1/vps1* strain (see Fig. 5A). Taken together, these data suggest that ploidy reduction can occur abruptly in a single step for strains defective in the *LAS21*, *GGA2* and *VPS1* genes. Our results indicate that genomic change as large as a ploidy shift may be caused by a single-gene deletion.

The FACS analysis also revealed other ploidy shift scenarios occurring in the cells with vesicular trafficking disorders. The analyzed strains showed signs of ploidies that were not expected. The strains bearing deletions of the *ARF1*, *PEP12*, *PEP7* and *VPS33* genes displayed a nearly triploid DNA content, whereas a lack of the *BFR1* gene led the strain to tetraploidy. The *VPS45*-deficient strain became polyploid, and the corresponding FACS histogram suggested significant DNA content variation within the *vps45/vps45* cell population. The deletion of the *ANP1*, *CAX4*, *GGA2*, *FEN1*, *NHX1*, *PEP3*, *RVS161*, *SWA2*, *VID22* and *VPS63* genes resulted in a shift of the peaks to positions typical of aneuploids. Moreover, certain analyzed strains presented cell-cycle abnormalities. For example, in the *vps3/vps3*, *arf1/arf1*, *pep12/pep12*, *pep7/pep7* or *vps45/vps45* strains, an overrepresentation of S phase cells was visible, even in asynchronous cell populations, which reflects prolonged persistence in this phase of the cell cycle. The shift in ploidy observed in these strains was likely caused by replication stress, which has also been suggested for human cells in which a prolonged replication block or harsh DNA damage that causes mitotic catastrophe at a certain frequency ends with abnormal cell division and leads to aneuploidization or polyploidization through the failure of cytokinesis or mitotic slippage [2]. Increased ploidies have primarily been reported for strains in which the deletion of a certain gene or its mutation affects histone deposition, which in turn influences chromatin condensation or relaxation and affects different processes connected to DNA replication and repair and/or chromosome segregation [95–97]. The presence of a genome instability phenotype related to genes involved in vesicular trafficking is novel and puzzling. However, there are a few reports concerning the involvement of genes related to vesicular trafficking in DNA maintenance processes, such as the homeostasis of telomere length [52,98] or DSB repair of broken chromosome ends [99]. The latter study revealed the involvement of the *VID22* gene, which was also identified in our zeocin oversensitivity screen in the promotion of efficient DNA repair through the influence of the chromatin structure at the damage site via nucleosome eviction around the DSB. Thus, inactivation of Vid22 impairs break repair, leading to an easily detectable abnormal FACS profile (S4 Fig. A).

### Strains harboring gene deletions impairing vesicular trafficking are unable to repair zeocin-initiated damage

To verify the engagement of vesicular trafficking-related genes in the cellular response to DSB stress, we tested whether the corresponding deletion strains were able to recover from zeocin stress. Chromosomes isolated from cells that were untreated, treated with zeocin, or allowed to recover following zeocin treatment were resolved via pulsed-field gel electrophoresis (PFGE). The following strains belonging to different categories with respect to their DNA content were chosen for this experiment: *vps1/vps1* from the haploid category; *nhx1/nhx1*, *vid22/vid22* and *vps63/vps63* from the aneuploid category; *gga2/gga2* from the aneuploid category with an intriguing sub-G1 population; *arf1/arf1* and *pep12/pep12* from the triploid category; and finally *vps33/vps33* from the triploid category with a tendency to undergo a further increase in ploidy.

For this experiment, we constructed *de novo* diploid deletion strains to avoid possible secondary changes that might have accumulated in potentially genetically unstable strains during the long YKO collection history. Because a particular gene disruption can affect genome stability and cause a rapid accumulation of secondary changes, it is not possible to obtain an exactly diploid DNA content in such strains. The results obtained for the freshly prepared strains in the FACS assays are presented in Fig. 5A. It was possible to obtain nearly diploid strains lacking the *GGA2*, *NHX1*, *VID22* and *VPS51* genes; these strains occurred in the YKO diploid collection as aneuploids except for the strain lacking *VPS51*, which occurred as a haploid. The strain harboring a deletion of the *VPS1* gene, however, showed a rapid reduction of its DNA content to a haploid level. Moreover, half of the *vps1/vps1* clones examined during the construction process transformed into haploids so quickly that we were unable to pinpoint the occurrence of this event (data not shown). The haploidization process was even faster in the *vps63/vps63* strain. The DNA contents of the freshly prepared strains harboring deletions of the *VPS45*, *VPS3*, *ARF1* and *PEP12* genes were not normal, yet they did not accumulate as many secondary changes as did their equivalent strains from the diploid YKO collection. In all of the subsequent experiments, we used strains prepared in our laboratory.

A PFGE analysis was employed to examine the extent to which the chromosomes of freshly prepared homozygous diploids were affected by zeocin-induced damage and measure the ability of particular strains to repair DNA damage caused by this agent. The assay was performed in exponentially growing cells because the most severe effect of this drug is expected to occur in rapidly growing and dividing cells. We analyzed genomic DNA isolated from the cells treated with (1) nocodazole (15 μg/ml) to synchronize cells in mitosis when the chromosomes were fully replicated; (2) zeocin (150 μg/ml) to detect the extent of damage; and (3) zeocin, which were then washed and permitted to recover for 3 hours in medium supplemented with nocodazole. This type of treatment provides cells the time to repair damage and prepare chromosomes for the next division. As shown in Fig. 5B, although the WT strain was proficient in DNA repair after zeocin treatment, none of the analyzed strains was able to recover. All of the deletion strains included in the analysis displayed the same inability to recover as the two positive control strains with homozygous deletions of the *XRS2* and *RAD52* genes, which are involved in DNA damage recognition and repair, respectively. Because none of genes involved in NHEJ appeared in our zeocin oversensitivity screen, we also included the *yku70/yku70* strain as a negative control in our PFGE experiment. As expected, the strain lacking functional NHEJ showed full recovery from the DSB stress initiated by zeocin (see Fig. 5B).

### Strains with impaired vesicular trafficking display altered formation of Rad52-YFP foci

Homologous recombination is the major pathway responsible for repairing DSBs, and the Rad52 protein plays a pivotal role in this pathway by facilitating Rad51 loading onto RPA-coated ssDNA and seeking out and mediating the annealing of homologous DNA strands [100]. Rad52 has been shown to be recruited to damaged sites in DNA where it forms recombinant centers, and these centers can be visualized via fluorescent microscopy using gene fusion that encodes *Rad52* fused to a fluorescent protein [101]. Therefore, it is possible to diagnose DNA integrity *in vivo* using Rad52 as a marker by counting fluorescent Rad52 foci [102].

We employed a Rad52-YFP fusion [103] to estimate the repair ability of strains with impaired vesicular trafficking. Our data showed that the number of foci per cell did not vary greatly across the strains and applied conditions; however, there were more cells with Rad52 foci in the deletion strains than in the WT strain (see Fig. 5C-E). The strains lacking the *VPS3*, *VPS45*, *VPS51*, *VPS63*, *VID22*, *GGA2*, *NHX1* and *PEP12* genes displayed up to threefold increases in the spontaneous formation of Rad52 foci compared with the WT strain, whereas the strains lacking *ARF1*, *VPS1*, *VPS3*, *VPS63*, *VID22*, *NHX1* and *PEP12* displayed up to threefold increases in the Rad52-YFP foci after zeocin treatment compared with the WT strain under the same conditions. The same rate of increased Rad52 focus formation was previously reported for strains defective in either DNA replication and repair or DNA damage checkpoints [102]. Interestingly, the strain lacking the *VPS51* gene exhibited the highest level of spontaneous formation of Rad52 foci among all of the analyzed strains. The number of spontaneously formed Rad52 foci in the *vps51* cells was equivalent to the number formed in response to zeocin exposure in the WT strain. However, the number of Rad52 foci in this strain did not increase following additional zeocin treatment. It has recently been shown that Vps51 is required for adaptation to the DNA damage checkpoint [104]. During G2/M arrest after DNA damage, transient activation of the cytoplasm-to-vacuole-mediated degradation of securin Pds1 and nuclear exclusion of separin Esp1 are observed, which allows for the resumption of mitosis. In the strains with mutations in components of the GARP complex, such as the strain lacking *VPS51*, no recovery is observed. In the *vps51* strain, the checkpoint is permanently activated because of increased autophagy, which causes partial degradation of Pds1 [104]. Therefore, the observed elevated level of Rad52 foci in the *vps1* strain might be explained by checkpoint activation, and a further increase in the number of foci should not be expected.

What might be the reason for the increased number of Rad52 foci in the other analyzed strains? (1) Such an increase could reflect an increased number of DNA lesions, or (2) it might have been caused by foci persisting over time because of altered kinetics of the assembly/disassembly of foci or alterations in DNA repair. The first explanation would be valid for the *VPS63*-deleted strain, which was shown to accumulate zeocin, and may be valid for the *ARF1*-deleted strain, in which the endogenous ROS level was elevated. In that strain, oxidative stress and zeocin contributed to DNA damage. However, the methods by which Rad52 foci occur in the other zeocin-oversensitive strains with impaired vesicular trafficking are unclear.

### Various vesicular trafficking pathways are involved in the cellular response to genotoxic stress and influence genome stability in diverse ways

**Fig 6:**
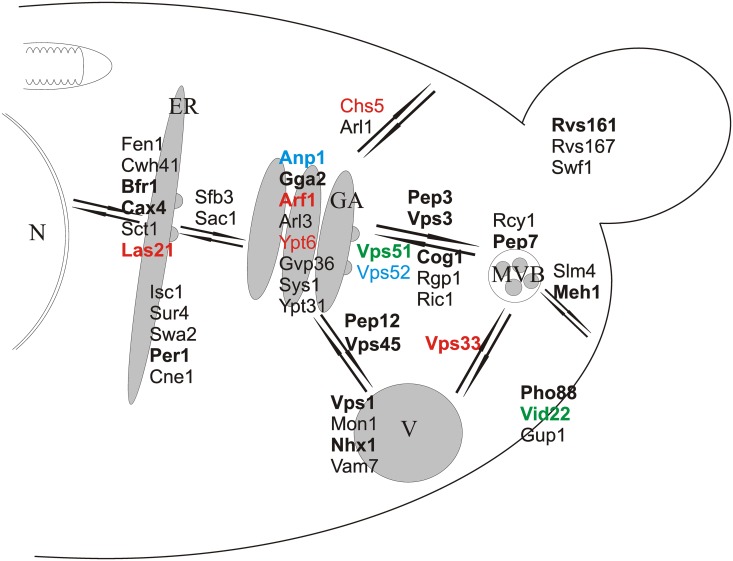
Proteins involved in vesicular trafficking and in protection against zeocin-mediated damage. Proteins were placed in the scheme according to their function in a particular vesicular trafficking sub-pathway. In the case of proteins participating in multiple sub-pathways, only one localization is shown in the scheme for clarity. Genes with a documented role in genome maintenance are indicated in green. Genes preventing zeocin accumulation are indicated in blue. Genes that are required to reduce intracellular ROS levels are indicated in red. Genes required for maintaining proper DNA content are indicated in bold font. Abbreviations: N—nucleus, ER—endoplasmic reticulum, GA—Golgi apparatus, V—vacuole, MVB—multi-vesicular body.

Our experiments showed that the impairment of various vesicular trafficking pathways affects different aspects of the zeocin stress response and lead to oversensitivity of this agent via different mechanisms (see Fig. 6). The link between genome stability and vesicular trafficking may initially seem surprising; however, traces of this link can be found in the literature, although it has generally been considered to be coincidental rather than consequential [105,106]. In addition to our zeocin sensitivity analysis, three other genome-wide approaches have revealed a substantial number of vesicular trafficking genes whose deletion results in sensitivity to bleomycin [81] or genotoxin CdtB (cytolethal distending toxin subunit B, the virulence protein secreted by certain bacterial pathogens) [107] or leads to the presence of a dysfunctional mutated topoisomerase I, *top1-T*_*722*_ *A*, in the cell [69]. In all of these instances, the damaging agent acts mostly by generating breaks in DNA. We performed a comparison of the results of all four studies in the form of a Venn diagram (S5 Fig.) and concluded that the sizes of the intersections were rather small. Only a few overlapping genes were found between these experiments, with as few as 4 genes appearing in all of them: *CTF8*, *RAD57*, *XRS2* and *ZUO1*. There was even less overlap of vesicular trafficking genes indicated in these studies [69,81,107]. Because all of these screens test for sensitivity to DNA breaks, it is unclear why the overlaps are so small, although there are several possible explanations. First, these experiments were conducted using different yeast strain collections: a haploid gene deletion collection was employed for the *top1-T*_*722*_ *A* overexpression and bleomycin treatment experiments; non-essential homozygous deletion diploids were utilized for the CdtB expression experiment; and homozygous diploids for non-essential genes and heterozygous diploids for essential genes were employed in our zeocin sensitivity screen. Second, the lesions introduced into DNA by the damaging agents used in these experiments are different. The radiomimetic compounds bleomycin and zeocin predominantly provoke DSBs, whereas the genotoxin CdtB causes SSBs, and the effect of *top1-T*_*722*_ *A* mimics that of camptothecin, which is a drug that traps topoisomerase I at the ends of cut DNA. The heterogeneity of the results of these screens suggests the occurrence of cellular mechanisms that can distinguish between various types of DNA damage and react accordingly, recruiting appropriate cellular pathways to fix the problem.

Although vesicular trafficking genes were detected in all three of the cited genome-wide studies, the authors demonstrated the role of selected genes in DNA maintenance rather than the systematic contribution of vesicular trafficking to genome stability. The authors revealed the involvement of the L-carnitine transporter Agp2 and kinases regulating polyamine transport (Ptk2 and Sky1) in bleomycin uptake [81] as well as the participation of Vps25 (an ESCRT complex component involved in protein targeting to the vacuole) in Top1 degradation [69]. The importance of vesicular trafficking to the vacuole was also noted by Kitagawa et al. [107]. These authors observed susceptibility of several ESCRT III complex and vacuole mutants to hydroxyurea and Ho overexpression and noted an increase in the DNA contents of these mutant cells. However, the authors only speculated that these phenotypes might be caused by the accumulation of incomplete mRNAs, which would lead to the production of aberrant proteins and their accumulation in the described mutants [107].

Therefore, to our knowledge, the present report describes the first systematic approach for verifying vesicular trafficking-deficient mutants with phenotypes that present DNA instability. Our data demonstrate the importance of vesicular trafficking for the maintenance of genomic DNA. It appears that proficient vesicular trafficking is crucial for the efficient repair of spontaneous and zeocin-mediated DNA damage and proper functioning of the HR pathway. We demonstrated that the deletion of genes involved in vesicular trafficking frequently leads to DNA content anomalies. Further studies will be required to determine how vesicular transport influences genome stability mechanisms. Nevertheless, certain conclusions can already be drawn from our results. We showed that the *ANP1* and *VPS52* genes are necessary to reduce intracellular level of zeocin. Additionally, we demonstrated that the *LAS21*, *VPS33*, *ARF1*, *YPT6* and *CHS5* genes contribute to ROS homeostasis, suggesting that the genome becomes unstable because of the increase in intracellular ROS as a secondary effect of general cell dysregulation caused by the impairment of vesicular trafficking. However, for the majority of vesicular trafficking-related genes revealed in our screen, the involvement of their protein products in genome maintenance processes remains unknown.

Fig. 6 provides a schematic overview of vesicular trafficking pathways in which the proteins involved in protection against zeocin-provoked damage are inserted and shown to participate in traffic in almost all directions. Moreover, the proteins whose absence leads to DNA content anomalies and who act as guardians of genome stability are present in every branch of traffic, indicating the multiple roles these proteins play in genome maintenance processes. Taken together, our results clearly show that vesicular trafficking and the various involved pathways are essential for genome maintenance and provide various contributions to this vital cellular function.

## Materials and Methods

### Strains and plasmids

The *S*. *cerevisiae* yeast gene knock-out collection version 2 (YKO) that was created for the *Saccharomyces* Genome Deletion Project [34] was obtained from Open Biosystems (Huntsville, AL, USA) as deep-frozen glycerol stocks in 96-well microtiter plates. Diploid pools of the YKO strains were prepared as previously described [3].

Unless stated otherwise, all experiments were performed in the BY474X background: BY4741 (*MAT*a *his3Δ leu2Δ ura3Δ met15Δ*), BY4742 (*MAT*α *his3Δ leu2Δ ura3Δ lys2Δ*) and BY4743 *(MATa/alpha his3Δ/his3Δ leu2Δ/leu2Δ ura3Δ/ura3Δ LYS2/lys2Δ MET15/met15Δ*) or its derivatives: 1n (*MAT*a *his3Δ leu2Δ met15Δ*), αC:L (*MAT*α *his3Δ leu2Δ ura3Δ lys2Δ can1*::*LEU2*), and 2n *(MATa/alpha his3Δ/his3Δ leu2Δ/leu2Δ URA3/ura3Δ CAN1/can1*::*LEU2 LYS2/lys2Δ MET15/met15Δ*) [3].

The diploid strains prepared *de novo* for DNA instability phenotype verification are listed in S1 Table.

The haploid strains were investigated in the BY474X derivative background using the gene replacement method with a disruption cassette amplified via PCR with primers designed specifically for each construct (S2 Table) and genomic DNA from the respective YKO clone as a template. The correctness of the gene disruptions was verified via PCR. After the introduction of a particular gene deletion into the haploid strains of both mating types, subsequent clones were mated to obtain a diploid strain bearing a double deletion. Strain YAS330 (*rad52*::*HIS3/rad52*::*HIS3*) was prepared similarly except that the deletion cassette was amplified using pRS313 as a template with the long primers Rad52.kup and Rad52.klw, which show 50 bp of homology to the upstream and downstream region of the *RAD52* gene, respectively. For the analysis of Rad52-YFP foci, the plasmid pWJ1344 [103] was used, which was kindly provided by Dr. Michael Lisby.

### Culture media and growth conditions

The rich YPD medium contained 1% yeast extract (Difco, Mt. Pritchard, NSW, Australia), 2% bactopeptone (Difco) and 2% glucose (POCh, Gliwice, Poland). The YPD-GPS medium was YPD medium supplemented with G-418 (100 μg per ml; Calbiochem, Darmstadt, Germany), penicillin (50 μg per ml; Polfa Tarchomin S.A., Poland) and streptomycin (50 μg per ml; Sigma-Aldrich, St. Louis, MO, USA). The SC medium contained 0.67% Yeast Nitrogen Base (Difco) and 2% glucose and was supplemented with all amino acids, uracil and adenine (Formedium, Hunstanton, UK). The minimal yeast nitrogen base medium contained 0.67% Yeast Nitrogen Base (Difco) and 2% glucose. The solid medium also contained 2.5% agar (Difco).

Unless stated otherwise, liquid cultures were grown at 28°C with agitation at ~ 200 RPM (New Brunswick Scientific, Edison, NJ, USA). Growth on solid media was performed at 28°C. The media were sterilized using microwave technology with an EnbioJet microwave autoclave (Enbio Technology, Kosakowa, Poland).

### High-throughput zeocin sensitivity screen

Pools of the *S*. *cerevisiae* YKO strains from the homozygous diploid and heterozygous essential gene collections were inoculated into a single flask with YPD medium and cultured at 28°C with shaking to a density of approximately 1–2x10^7^ cells/ml. The culture was then split into three subcultures, with one used as the untreated control and the other two supplemented with 5 or 15 μg per ml zeocin (InvivoGen, San Diego, CA, USA) and grown in parallel for one hour. The cells from each subculture were then harvested, washed and plated on five large (diameter 150 mm) SC plates at 5x10^6^ cells per plate. The plates were incubated at 28°C for three days. The colonies were then washed off the plates for genomic DNA isolation and subsequent barcode analysis. Four biological replicates were performed.

### DNA labeling and hybridization to Agilent Barcode Arrays

Genomic DNA was isolated from each sample (10^9^ cells), and the barcodes were labeled via PCR using fluorescently labeled primers and hybridized to custom-made barcode microarrays (Agilent, Santa Clara, CA, USA). Scanning and feature extraction were performed using an Axon GenePix 4000B scanner and GenePix Pro software (Molecular Devices, Sunnyvale, CA, USA). For the detailed protocols, see our previous work [3].

### Data analysis

A statistical analysis of the data was performed using Acuity 4.0 (Molecular Devices). Certain operations were also conducted in Excel 2007 (Microsoft, Redmond, WA, USA). For each deletion clone pool and zeocin dosage, eight raw data files were obtained (two biological replicates, each in two technical replicates with dye-swapping and separate data files for UP-TAG and DOWN-TAG barcodes). Ratio-normalized raw LogRatio data were averaged within each group, resulting in four datasets. The deletion clones that were significantly underrepresented after zeocin treatment (LogRatio <-1, p-value <0.1) according to at least one of the four datasets were considered to be zeocin oversensitive, and the respective genes were included in the initial list of 166 genes, which were subsequently subjected to individual verification.

### Data deposition

The microarray datasets supporting the results of this study are Minimum Information About a Microarray Experiment (MIAME) compliant and available in the Gene Expression Omnibus repository (GEO) under accession number GSE59193 [108].

### Individual tests: the sensitivity drop assay

To estimate the level of sensitivity to zeocin in individual tests, we performed a semi-quantitative drop-assay. Each deletion strain was grown in YPD medium with shaking at 28°C to a density of approximately 1–2x10^7^cells per ml. The cells were then spun down, washed with 0.9% NaCl (POCh), and adjusted to a density of 3.3x10^7^ cells per ml via resuspension in the same solution. Five 6-fold serial dilutions in a 0.9% NaCl solution were performed for each strain, and 3.3 μl of each dilution was spotted onto Omnitray (Nunc, Roskilde, Denmark) plates containing selection medium (YPD with 2.5 μg per ml zeocin; 5 μg per ml zeocin; 5 μg per ml zeocin and 50 mM KCl (POCh); or 50 mM KCl alone), or YPD medium for the dilution control. The plates were then incubated at 28°C for 2 days. The countable spots of the colonies grown on all types of plates were totaled, and survival on zeocin was calculated, taking the dilution factor into account.

### Individual tests: the quantitative zeocin sensitivity test

To verify the zeocin sensitivity phenotype of strains that were slightly sensitive in the drop sensitivity assay, a more accurate measurement of survival rate after zeocin treatment was performed. Each deletion strain was grown in YPD medium with shaking at 28°C to a density of approximately 1–2 x 10^7^cells per ml. The cells were then spun down, washed with 0.9% NaCl, and adjusted to a density of 3000 cells per ml via resuspension in the same solution. The 100 μl of cell suspension (approximately 300 cells) was plated in duplicates on plates containing selection medium (YPD with 2.5 μg per ml zeocin or 5 μg per ml zeocin) or YPD medium as the control. The plates were then incubated at 28°C for 2 days. The colonies grown on all types of plates were counted, and the survival on zeocin was calculated. The survival rate (percent of clones growing on selective media in respect to clones growing on control plates) was calculated as the average from at least 5 (up to 10) independent cultures of each strain.

### FACS analysis

The DNA content of *S*. *cerevisiae* cells was measured via flow cytometry as previously described [3]. Briefly, the cells from 1 ml of a yeast exponential culture (OD_600_ ~0.2–0.5) were spun down and subjected to permeabilization and fixation via suspension in 1 ml of a chilled (-20°C) 70% ethanol (Polmos, Warsaw, Poland) solution. The suspensions were held at RT for at least 2 hours. The fixed cells were then washed twice in FACS buffer (0.2 M Tris-HCl (Sigma-Aldrich), pH 7.4, 20 mM EDTA (Merck, Darmstadt, Germany) and incubated for 2 hours at 37°C in FACS buffer with 1 mg per ml RNase A (Sigma-Aldrich) to eliminate RNA. The cells were then washed with phosphate-buffered saline (PBS) and stained overnight at 4°C in the dark with 100 μl of propidium iodide solution (50 μg per ml in PBS; Calbiochem). After the addition of 900 μl PBS, the cells were vortexed vigorously prior to FACS analysis of the DNA content, which was performed using a FACSCalibur analyzer (Becton-Dickinson, Franklin Lakes, NJ, USA). A total of 10,000 cells were counted in a single assay.

### Recovery after zeocin treatment assay

Diploid yeast strains were cultured in YPD medium at 28°C with shaking to a density of approximately 1–2x10^7^ cells per ml. Each culture was then divided into two parallel subcultures in a 2:1 proportion. The smaller subculture, which served as a control, was supplemented with 15 μg per ml nocodazole (Sigma-Aldrich) and incubated for 3 hours at 28°C with shaking. The larger subculture was supplemented with 150 μg per ml zeocin and incubated for one hour at 28°C with shaking. The cells were then spun down, washed twice and resuspended in fresh YPD, and the suspension was divided again into two subcultures of equal volume. One of the subcultures served as a zeocin-treated probe, and 15 μg per ml nocodazole was added to the other, after which the cells were incubated for additional 3 hours at 28°C with shaking to allow for recovery from genotoxic stress. Precisely 10^7^ cells from each of the three cultures were used for PFGE of their chromosome integrity.

### Pulsed-field gel electrophoresis of yeast chromosomes

The analysis of yeast chromosome sizes was performed as previously described [109] with certain modifications. Yeast cells were embedded in 20 μl plugs of low melting point InCert agarose (Lonza, Basel, Switzerland) and digested with Zymolyase 100T (BioShop, Burlington, ON, Canada), followed by proteinase K (Sigma-Aldrich) and RNase A at 30°C with gentle rotation (4 rpm) using rotator SB3 (Bibby Sterlin LTD, Stone, UK). The released genomic DNA was separated in a CHEF Mapper III Pulsed Field Electrophoresis System (BioRad, Hercules, CA, USA) for 22 h at 5.9–6 V per cm and 12°C, with the angle set to 120°, switch time set to 60 s and 80 s and ramping set to 0.8. The separated chromosomes were stained with ethidium bromide (Sigma-Aldrich), which was illuminated with 302 nm UV light and digitized with a charge-coupled device camera (Fluorchem Q Multi Image III, Alpha Innotech, San Leandro, CA, USA).

### Measurement of cellular ROS

ROS detection was performed as previously described [110] with minor modifications. Briefly, the yeast strains were cultured overnight in YPD medium at 28°C with shaking to post-diauxic phase (approximately 1.5–2x10^8^ cells per ml). Then, 10^8^ cells from each culture were collected, washed twice and resuspended in 1.5 ml of distilled H_2_O. 2,7-dichloro-dihydro-fluorescein diacetate (DCFH-DA, Sigma-Aldrich) was added to a final concentration of 10 μM from a 5 mM ethanol stock, and the cell suspensions were incubated for 30 minutes at 30°C. Cells from each sample were spun down, washed twice with water, flash-frozen in liquid nitrogen and disrupted with 0.3 g of glass beads in 1.5 ml of STET buffer (10 mM Tris—HCl, pH 8.0, buffer containing 1% sodium dodecyl sulfate (Biomol, Enzo Life Sciences, Farmingdale, NY, USA), 2% Triton X-100 (BDH Chemicals, Poole, UK), 100 mM NaCl, and 1 mM EDTA) via vigorous vortexing for 10 min. After an additional 10 min incubation at room temperature, the cell debris was spun down, and the fluorescence of the supernatant was measured using a Cary Eclipse fluorescence spectrophotometer (Varian, Palo Alto, CA, USA), with fluorescence excitation at 485 nm and emission at 522 nm. The ROS levels in relative fluorescence units were calculated as an average (with the standard deviation) from the data of at least four independent cultures of each strain.

### Radiolabeling of zeocin

First, 10 mg of zeocin was dissolved in 100 μl of tritiated water (Moravek, Brea, CA, USA; specific activity 5 Ci (185 GBq) per g) and maintained at room temperature for 17 h in the dark. Then, labile tritium was removed through several rounds of vacuum evaporation with 5 ml of a toluene:acetone (1:1) mixture in a Buchi rotary evaporator (Model RE 114; Buchi, Flawil, Switzerland) at bath temperatures of less than 37°C. The resulting specific activity of the product was approximately 0.16 mCi (6 MBq per g)

### Measurement of intracellular ^3^H-zeocin levels

To estimate the cellular levels of zeocin in the individual strains, we used radiolabeled zeocin. Each deletion strain was grown in YPD medium with shaking at 28°C to a density of approximately 1–2x10^7^ cells per ml. All cell suspensions were brought to the same density of 2x10^7^ cells per ml, and ^3^H-zeocin was added to 12 ml of the cell suspension at a final concentration of 15 μg per ml. After one additional hour of incubation, the cells from individual cultures were collected via vacuum filtration on glass fiber filters (Whatman GF/C, GE Healthcare, Little Chalfont, UK) and washed twice with 5 ml of water. The dried filters were placed in scintillation vials with 3.5 ml of scintillator cocktail (PPO/POPOP in toluene), (PPO (Roanal, Budapest, Hungary), POPOP (Koch-Light, Colnbrook, UK), toluene (POCh)), and radioactivity was measured using a PerkinElmer Tri-Carb 2910TR liquid scintillation analyzer (Perkin Elmer, Waltham, MA, USA). After subtraction of background radioactivity (the filtrate from ^3^H-zeocin-containing medium without cells), the readout from all replicates (at least four) for each strain was averaged, and the standard deviation was calculated.

### Detection of Rad52-YFP foci via fluorescence microscopy

To determine the number of Rad52 foci, deletion strains in the BY4741 background were used because recombination foci remain for longer periods in haploid strains because of the increased time required for homology searches. The yeast strains were transformed with the plasmid pWJ1344 carrying a RAD52-YFP fusion [103]. The transformants were grown in SC-LEU medium in 28°C with shaking until the exponential phase. An aliquot of each culture was collected for assessment of the level of spontaneous formation of Rad52 foci. The remaining culture was treated with 100 μg per ml zeocin for one hour, and the zeocin-induced Rad52 foci were then quantified. The cells were examined under a fluorescence microscope (Axio Imager.M2, Zeiss, Oberkochen, Germany) using a 46HE filter set and under white light using Nomarski optics. The number of cells and Rad52-YFP foci in the cells were counted, and the percentage of cells with Rad52-YFP foci and the average number of Rad52-YFP foci per cell were calculated after screening of at least 600 cells.

## Supporting Information

S1 Fig An example of the zeocin sensitivity drop assay results showing various categories of sensitivity phenotypes. Zeocin-sensitive strains were divided into subcategories with respect to the strength of their sensitivity phenotype and the level of phenotype suppression by KCl. Cell suspensions were serially diluted and spotted onto selective plates with 5 μg/ml zeocin, 50 mM KCl or both compounds, and they were also spotted onto dilution control plates as described in the Materials and Methods.
(PDF)

S2 Fig The results of the quantitative zeocin sensitivity test. The 30 deletion strains showing the slightly sensitive phenotype in the drop sensitivity assay were analyzed using a quantitative test of zeocin sensitivity. The 27 strains confirmed the zeocin oversensitivity phenotype. The experiment showed different phenotypes of analyzed strains. Some strains showed sensitivity to a higher dose of zeocin only (curves marked in green). Some strains exhibited smaller colony size under selective conditions (e.g., *gvp36/gvp36* strain (A); curves marked with dotted lines). Three strains did not confirm the zeocin sensitivity phenotype (curves marked in blue).
(PDF)

S3 Fig Overrepresentation of GO annotations within the group of genes selected in the zeocin sensitivity screen. The analysis was performed using the BINGO plug-in and Cytoscape [41] with p-value<0.05. GO annotations pertaining to genome maintenance are indicated with dark gray, and those related to vesicular trafficking are indicated with medium gray. All other GO annotations are indicated with light gray. (A) Overrepresentation of biological process annotations. (B) Overrepresentation of molecular function annotations. (C) Overrepresentation of cellular component annotations. Annotations pertaining to the nucleus are shown in dark gray, whereas those pertaining to vesicles are shown in medium gray.
(PDF)

S4 Fig Phenotypic analysis of vesicular trafficking-impaired homodiploid deletion strains that are sensitive to zeocin. (A) Cellular DNA contents of the deletion strains in the BY4743 background. Propidium iodide-stained cells were analyzed via FACS as described in the Materials and Methods. The WT strains, BY4741 and BY4743, served as DNA content controls. (B) Intracellular levels of ^3^H-zeocin. Deletion strains from the diploid YKO collection in the BY4743 background or their newly prepared equivalents in a 2n background were treated with 15 μg per ml of ^3^H-zeocin for one hour followed by measurement of the intracellular levels of ^3^H-zeocin (see the Materials and Methods for details). The BY4743 (WT), *rad52/rad52* and *yku70*/*yku70* strains served as controls. The histogram shows the average, standard deviation and median calculated from the CPM measurements from at least four independent samples for each strain. (C) Intracellular endogenous ROS levels in exponentially grown diploid YKO collection strains in the BY4743 background or their newly prepared equivalents in the 2n background. The BY4743 (WT) and the *sod1/sod1* strains served as controls for the WT and elevated ROS levels, respectively. The data for the *rad52/rad52*, *rad55/rad55*, *xrs2/xrs2* and *yku70*/*yku70* strains defective in DNA repair are shown for comparison. Intracellular ROS levels were determined fluorometrically with DCFH-DA as described in the Materials and Methods. Relative fluorescence units were normalized to the number of cells used in the assay. The histogram shows the average and median of at least four independent measurements for each strain, and the error bars represent the standard deviation.
(PDF)

S5 Fig Venn diagram showing comparison of four genome-wide screens performed to identify genes responsible for surviving under genotoxic stress. zeo—zeocin sensitivity screen (this work), top1-T_722_A—toxic allele of topoisomerase I sensitivity screen [1], bleo—bleomycin sensitivity screen [2] and CdtB—genotoxin CdtB sensitivity screen [3]. Analysis was performed using on-line Venn diagram tool of the Bioinformatics & Evolutionary Genomics webpage (http://bioinformatics.psb.ugent.be/cgi-bin/liste/Venn/calculate_venn.htpl)
(PDF)

S1 Table Strains used in this study. (PDF)

S2 Table Primers used in this study. (PDF)
