# Correction: A Genomic Screen Revealing the Importance of Vesicular Trafficking Pathways in Genome Maintenance and Protection against Genotoxic Stress in Diploid *Saccharomyces cerevisiae* Cells

**DOI:** 10.1371/journal.pone.0124186

**Published:** 2015-04-07

**Authors:** The PLOS ONE Staff

The fourth author’s name is spelled incorrectly. The correct name is Maria Bretner. The correct citation is: Krol K, Brozda I, Skoneczny M, Bretner M, Skoneczna A (2015) A Genomic Screen Revealing the Importance of Vesicular Trafficking Pathways in Genome Maintenance and Protection against Genotoxic Stress in Diploid *Saccharomyces cerevisiae* Cells. PLoS ONE 10(3): e0120702. doi:10.1371/journal.pone.0120702. The journal apologizes for this error that was introduced during the typesetting process.
