# Supplementary material for: A Genomic Screen Revealing the Importance of Vesicular Trafficking Pathways in Genome Maintenance and Protection against Genotoxic Stress in Diploid Saccharomyces cerevisiae Cells
Source: PLoS One. 2015 Mar 10;10(3):e0120702. doi: 10.1371/journal.pone.0120702 (PMC4355298; doi:10.1371/journal.pone.0120702)
Supplement: S1 Fig — Zeocin-sensitive strains were divided into subcategories with respect to the strength of their sensitivity phenotype and the level of phenotype suppression by KCl. Cell suspensions were serially diluted and spotted onto selective plates with 5 μg/ml zeocin, 50 mM KCl or both compounds, and they were also spotted onto dilution control plates as described in the Materials and Methods. (PDF) [file pone.0120702.s001.pdf]

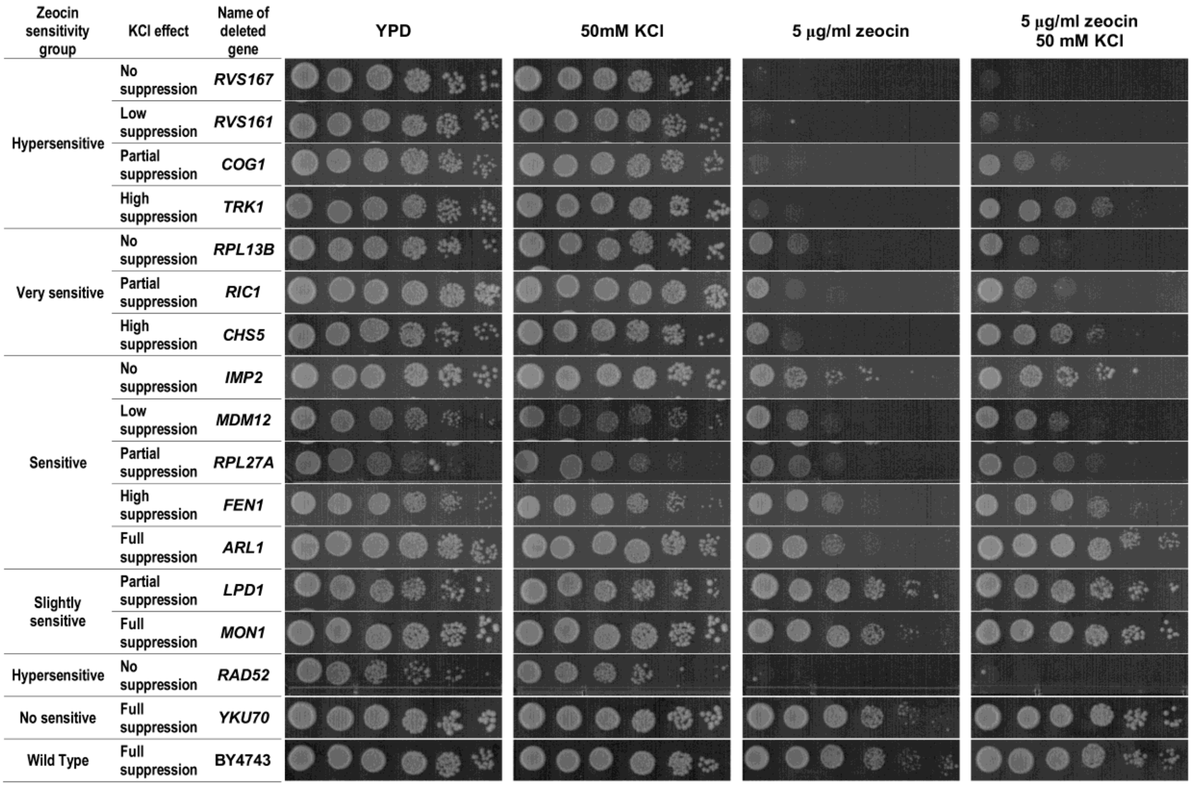

S1 Fig. An example of the zeocin sensitivity drop assay results showing various categories of sensitivity phenotypes. Zeocin-sensitive strains were divided into subcategories with respect to the strength of their sensitivity phenotype and the level of phenotype suppression by KCl. Cell suspensions were serially diluted and spotted onto selective plates with 5 µg/ml zeocin, 50 mM KCl or both compounds, and they were also spotted onto dilution control plates as described in the Materials and Methods.
