# Supplementary material for: A Genomic Screen Revealing the Importance of Vesicular Trafficking Pathways in Genome Maintenance and Protection against Genotoxic Stress in Diploid Saccharomyces cerevisiae Cells
Source: PLoS One. 2015 Mar 10;10(3):e0120702. doi: 10.1371/journal.pone.0120702 (PMC4355298; doi:10.1371/journal.pone.0120702)
Supplement: S2 Fig — The 30 deletion strains showing the slightly sensitive phenotype in the drop sensitivity assay were analyzed using a quantitative test of zeocin sensitivity. The 27 strains confirmed the zeocin oversensitivity phenotype. The experiment showed different phenotypes of analyzed strains. Some strains showed sensitivity to a higher dose of zeocin only (curves marked in green). Some strains exhibited smaller colony size under selective conditions (e.g., gvp36/gvp36 strain (A); curves marked with dotted lines). Three strains did not confirm the zeocin sensitivity phenotype (curves marked in blue). (PDF) [file pone.0120702.s002.pdf]

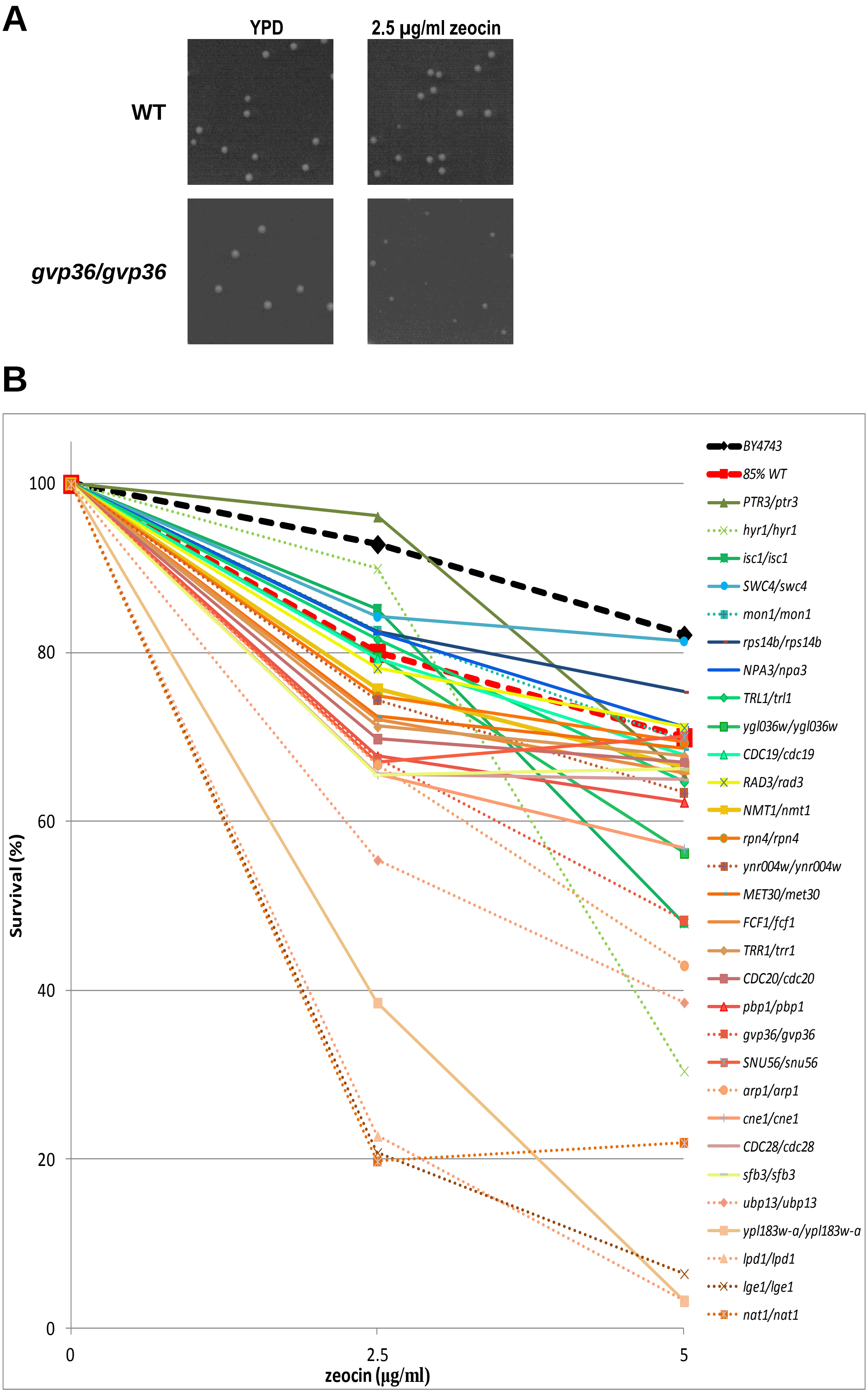

S2 Fig. The results of the quantitative zeocin sensitivity test. The 30 deletion strains showing the slightly sensitive phenotype in the drop sensitivity assay were analyzed using a quantitative test of zeocin sensitivity. The 27 strains confirmed the zeocin oversensitivity phenotype. The experiment showed different phenotypes of analyzed strains. Some strains showed sensitivity to a higher dose of zeocin only (curves marked in green). Some strains exhibited smaller colony size under selective conditions (e.g., *gvp36/gvp36* strain (A); curves marked with dotted lines). Three strains did not confirm the zeocin sensitivity phenotype (curves marked in blue).
