# Supplementary figures and images for: A Genomic Screen Revealing the Importance of Vesicular Trafficking Pathways in Genome Maintenance and Protection against Genotoxic Stress in Diploid Saccharomyces cerevisiae Cells
Source: PLoS One. 2015 Mar 10;10(3):e0120702. doi: 10.1371/journal.pone.0120702 (PMC4355298; doi:10.1371/journal.pone.0120702)

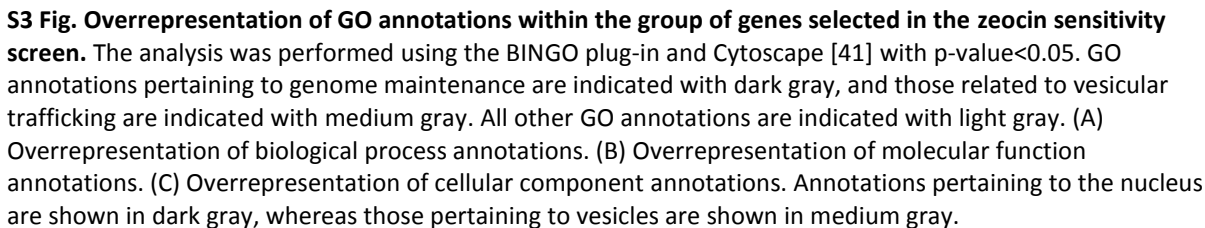

Supplement: S3 Fig — The analysis was performed using the BINGO plug-in and Cytoscape [41] with p-value<0.05. GO annotations pertaining to genome maintenance are indicated with dark gray, and those related to vesicular trafficking are indicated with medium gray. All other GO annotations are indicated with light gray. (A) Overrepresentation of biological process annotations. (B) Overrepresentation of molecular function annotations. (C) Overrepresentation of cellular component annotations. Annotations pertaining to the nucleus are shown in dark gray, whereas those pertaining to vesicles are shown in medium gray. (PDF) [file pone.0120702.s003.pdf]
