# Supplementary material for: A Genomic Screen Revealing the Importance of Vesicular Trafficking Pathways in Genome Maintenance and Protection against Genotoxic Stress in Diploid Saccharomyces cerevisiae Cells
Source: PLoS One. 2015 Mar 10;10(3):e0120702. doi: 10.1371/journal.pone.0120702 (PMC4355298; doi:10.1371/journal.pone.0120702)
Supplement: S4 Fig — (A) Cellular DNA contents of the deletion strains in the BY4743 background. Propidium iodide-stained cells were analyzed via FACS as described in the Materials and Methods. The WT strains, BY4741 and BY4743, served as DNA content controls. (B) Intracellular levels of 3H-zeocin. Deletion strains from the diploid YKO collection in the BY4743 background or their newly prepared equivalents in a 2n background were treated with 15 μg per ml of 3H-zeocin for one hour followed by measurement of the intracellular levels of 3H-zeocin (see the Materials and Methods for details). The BY4743 (WT), rad52/rad52 and yku70/yku70 strains served as controls. The histogram shows the average, standard deviation and median calculated from the CPM measurements from at least four independent samples for each strain. (C) Intracellular endogenous ROS levels in exponentially grown diploid YKO collection strains in the BY4743 background or their newly prepared equivalents in the 2n background. The BY4743 (WT) and the sod1/sod1 strains served as controls for the WT and elevated ROS levels, respectively. The data for the rad52/rad52, rad55/rad55, xrs2/xrs2 and yku70/yku70 strains defective in DNA repair are shown for comparison. Intracellular ROS levels were determined fluorometrically with DCFH-DA as described in the Materials and Methods. Relative fluorescence units were normalized to the number of cells used in the assay. The histogram shows the average and median of at least four independent measurements for each strain, and the error bars represent the standard deviation. (PDF) [file pone.0120702.s004.pdf]

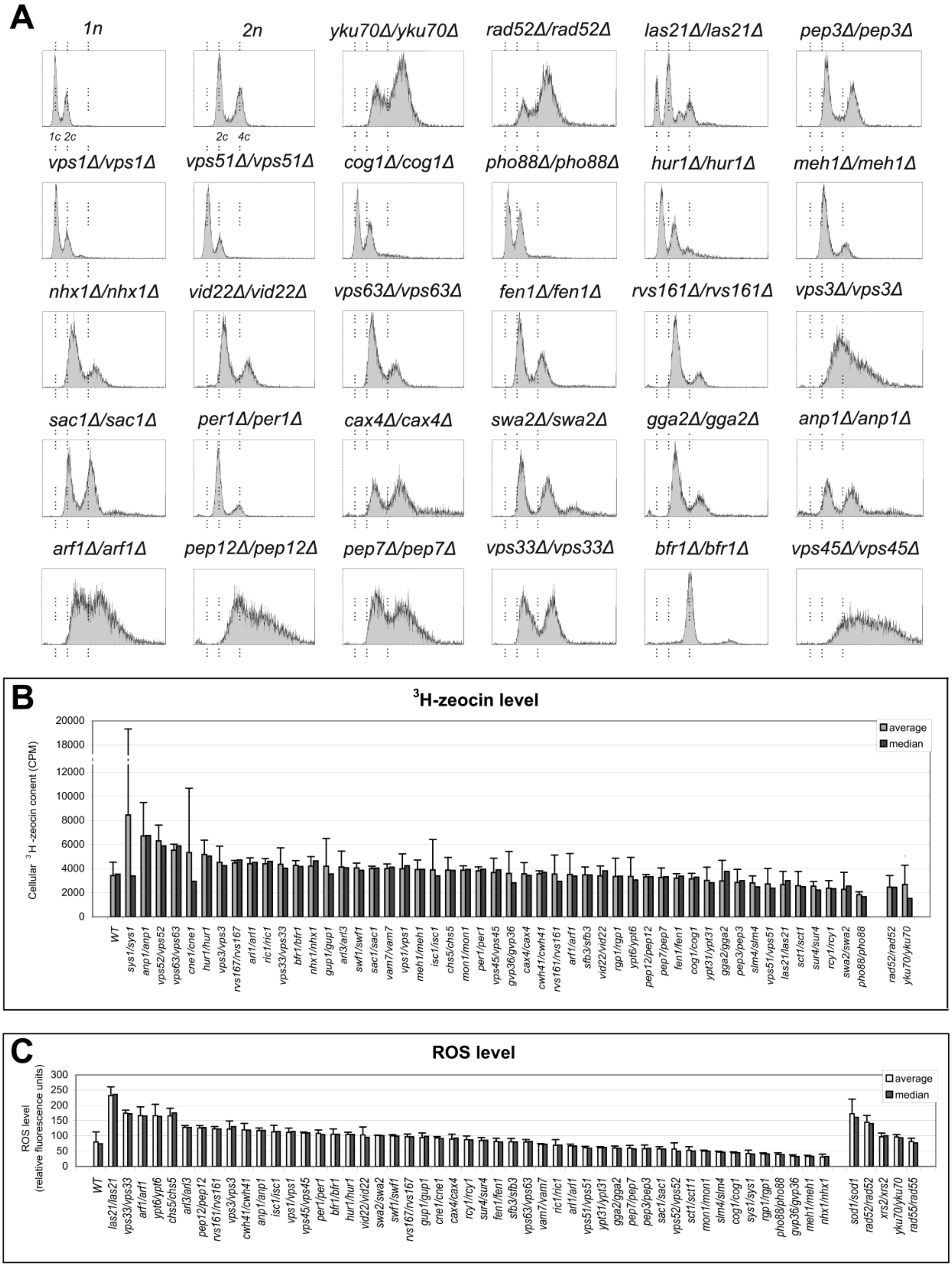

S4 Fig. Phenotypic analysis of vesicular trafficking-impaired homodiploid deletion strains that are sensitive to zeocin. (A) Cellular DNA contents of the deletion strains in the BY4743 background. Propidium iodide-stained cells were analyzed via FACS as described in the Materials and Methods. The WT strains, BY4741 and BY4743, served as DNA content controls. (B) Intracellular levels of ^{3}H-zeocin. Deletion strains from the diploid YKO collection in the BY4743 background or their newly prepared equivalents in a 2n background were treated with 15 µg per ml of ^{3}H-zeocin for one hour followed by measurement of the intracellular levels of ^{3}H-zeocin (see the Materials and Methods for details). The BY4743 (WT), *rad52/rad52* and *yku70*/*yku70* strains served as controls. The histogram shows the average, standard deviation and median calculated from the CPM measurements from at least four independent samples for each strain. (C) Intracellular endogenous ROS levels in exponentially grown diploid YKO collection strains in the BY4743 background or their newly prepared equivalents in the 2n background. The BY4743 (WT) and the *sod1/sod1* strains served as controls for the WT and elevated ROS levels, respectively. The data for the *rad52/rad52*, *rad55/rad55, xrs2/xrs2* and *yku70*/*yku70* strains defective in DNA repair are shown for comparison. Intracellular ROS levels were determined fluorometrically with DCFH-DA as described in the Materials and Methods. Relative fluorescence units were normalized to the number of cells used in the assay. The histogram shows the average and median of at least four independent measurements for each strain, and the error bars represent the standard deviation.
