# Supplementary material for: A Genomic Screen Revealing the Importance of Vesicular Trafficking Pathways in Genome Maintenance and Protection against Genotoxic Stress in Diploid Saccharomyces cerevisiae Cells
Source: PLoS One. 2015 Mar 10;10(3):e0120702. doi: 10.1371/journal.pone.0120702 (PMC4355298; doi:10.1371/journal.pone.0120702)
Supplement: S5 Fig — zeo—zeocin sensitivity screen (this work), top1-T722A—toxic allele of topoisomerase I sensitivity screen [1], bleo—bleomycin sensitivity screen [2] and CdtB—genotoxin CdtB sensitivity screen [3]. Analysis was performed using on-line Venn diagram tool of the Bioinformatics & Evolutionary Genomics webpage (http://bioinformatics.psb.ugent.be/cgi-bin/liste/Venn/calculate_venn.htpl) (PDF) [file pone.0120702.s005.pdf]

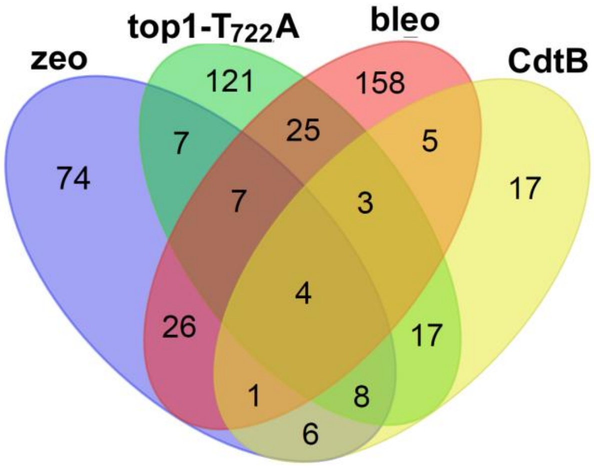

Screen	Total [1,2]	Gene name [3,4]
zeo top1 bleo CdtB	4	CTF8 RAD57 XRS2 ZUO1
zeo top1 bleo	7 (4)	RAD54 RIC1 RPL13B RVS167 SSZ1 VPS1 YPT6
zeo top1 CdtB	8 (1)	HUR1 RAD50 RAD51 RAD55 RAD59 MUS81 MMS4 SRS2
zeo bleo CdtB	1 (1)	RVS161
top1 bleo CdtB	3	ASF1 RAD27 RTT109
zeo top1	7 (1)	NUP60 RAD52 RGP1 SAM37 SOD1 TOP3 YDR455C
zeo bleo	26 (13)	ANP1 APQ13 BEM2 BFR1 CAX4 CNM67 ERG2 GUP1 HTL1 IMP2' NHX1 NPL6 PEP12 PLC1 RAD6 RCY1 ROT2 RPB9 RPL27A SRB5 SWF1 SWI6 VPS3 VPS45 VPS51 VPS52
zeo CdtB	6 (4)	PEP3 PEP7 PER1 SAC7 SGS1 SWA2
top1 bleo	25 (9)	BUD31 CCR4 CDC50 CTF18 CTF4 DIA2 DOC1 FAB1 GET2 GGC1 GLO3 MRE11 PMP3 POP2 RNR4 RSM7 SAC6 SHE4 SPT21 UGO1 VAM6 VPS20 VPS27 VPS8 VPS9
top1 CdtB	17	CTF19 DCC1 DDC1 EAF1 MCM16 MCM21 MCM22 MEC3 MMS1 RAD17 RAD24 RAD9 RTT101 RTT107 SAE2 TOF1 YLR235C
bleo CdtB	5 (2)	ASC1 DID4 NAT3 SPT10 VPS24
zeo	74 (25)	APL5 ARF1 ARL1 ARL3 ARP6 CDC19 CDC20 CDC28 CHS5 CNE1 COG1 CRD1 CWH41 DAL81 FCF1 FEN1 GGA2 GTR1 GVP36 HYR1 ISC1 LAS21 LGE1 LPD1 MDM12 MEH1 MET30 MON1 MSI1 NAT1 NMT1 NUP170 PBP1 PHO2 PHO88 PIN2 PIN4 PTR3 RAD3 RAD5 RPL12B RPL20A RPN4 SAC1 SCT1 SEH1 SFB3 SLM4 SNU56 SUR4 SYS1 TPN1 TRK1 TRL1 TRR1 TUP1 UBP13 VAM7 VID22 VPS33 VPS63 YBR194W YDL118W YDL119C YDR157W YDR524C-B YGL036W YGL057C YHR100C YHR151C YNL296W YNR004W YPL183W-A YPT31
top1	121 (25)	ARN1 ARO10 BRO1 BRP1 BSD2 CCH1 CIN5 CLB2 CLB5 CLN3 COQ10 CPA1 CSM3 DEP1 DUS4 FAA1 FRE4 FTR1 FUN30 FUR4 GRE2 HFD1 HMG2 HSP31 IMH1 IRC15 LSM6 LTE1 MET7 MMS2 MRPL11 MRPL22 MRPL28 MRPS9 MSH4 MVB12 NAM2 NCP1 NPT1 NTG2 NUP133 NUP84 OCA1 OMA1 PEP8 PEX5 PHO23 PKR1 PMP1 POR1 PPH3 PRY2 PSD2 PSY2 PTC1 PUB1 PXA2 QCR10 RAD61 RAS2 RAV2 RMI1 RPA34 RPD3 RPL20B RRF1 RRM3 RSM25 RUB1 SAP30 SIN3 SIS2 SKT5 SLA1 SLX4 SNF8 SPT2 SRC1 SRN2 STE50 STL1 STP22 TDP1 TEL1 TGL3 TNA1 URA5 VAM10 VAM3 VMA13 VMA5 VPS13 VPS17 VPS21 VPS28 VPS29 VPS35 VPS38 VPS5 VPS60 YAL064C-A YBP1 YBR099C YBR224W YER071C YGL214W YGR160W YGR205W YHR009C YHR159W YJR088C YJR116W YLR169W YLR294C YLR426W YML081W YML122C YMR291W YNL176C YOL048C YPR157W
bleo	158 (22)	ADE12 ADH1 ADK1 AFG3 AKR1 APL2 APM1 ARC1 ARC18 ARP5 ARV1 ATG17 ATP11 ATP12 ATP14 ATP15 BRE5 BUD16 BUD20 BUD23 BUD25 BUD27 BUD32 BUR2 CAT5 CDC10 CDC40 CHC1 CHS1 CTK1 CTK2 CTK3 CYS4 DBP7 DEF1 DHH1 DIA4 EAP1 EGD2 END3 ERG4 ERG6 EST2 FKS1 FUN12 FYV6 GAL11 GAS1 GCS1 GEM1 GET1 GLY1 GON7 GPH1 GRR1 HOC1 HOF1 IES6 ILM1 INP53 ISA1 IWR1 KCS1 KEM1 LAG2 LCB4 LOC1 MDJ1 MET22 MNN10 MNN9 MON2 MRPL51 MRPS8 MSF1 OCT1 OPI11 OPT2 OST4 PAP2 PAT1 PDA1 PEP5 PEX32 PFD1 PFK2 PHO85 PPA2 PRO1 RAI1 REG1 REI1 RIB4 RML2 RNR1 ROX3 RPB4 RPL1B RPL35A RPL39 RPP1A RPS0B RRN10 RSM19 RSM22 RTS1 SAC3 SFP1 SHP1 SIN4 SLG1 SLX8 SNF1 SNF6 SNT309 SPC72 SPF1 SPS4 SPT20 SPT7 SRB2 SRB8 SSN8 SSQ1 SWI4 SWS2 TAF14 THP1 TIF3 TIF4631 TOM5 TPS1 TUF1 UBP3 UMP1 VPH2 VPS15 VPS16 VPS25 VPS4 VPS54 VPS66 VPS69 YAF9 YCL007C YDJ1 YDR049W YDR532C YER087W YGL072C YGR237C YLR374C YME1 YMR031W-A YOR304C-A YOR333C YOR342C YPT7
CdtB	17 (2)	AFT1 BIM1 ELG1 CHL1 ELM1 MRC1 NAM7 NBP2 NMD2 NUP120 OPI9 PMR1 SNF7 UME6 UPF3 VPS65 YEL033W

[1] The number of vesicular trafficking genes involved in defense against zeocin toxicity are marked blue.
[2] Vesicular trafficking genes involved in defense against zeocin toxicity are marked in blue.
[3] The number of vesicular trafficking genes involved in defense against other stresses are marked green.
[4] Vesicular trafficking genes involved in defense against other stresses are marked in green.

S5 Fig. Venn diagram showing comparison of four genome-wide screens performed to identify genes responsible for surviving under genotoxic stress. zeo - zeocin sensitivity screen (this work), top1-T_{722}A - toxic allele of topoisomerase I sensitivity screen [1], bleo - bleomycin sensitivity screen [2] and CdtB - genotoxin CdtB sensitivity screen [3]. Analysis was performed using on-line Venn diagram tool of the Bioinformatics & Evolutionary Genomics webpage (http://bioinformatics.psb.ugent.be/cgi-bin/liste/Venn/calculate_venn.htpl)

1. Reid RJD, González-Barrera S, Sunjevaric I, Alvaro D, Ciccone S, Wagner M, Rothstein R: DNA damage protocol, identifies new genes affecting topoisomerase I -induced Selective ploidy ablation, a high-throughput plasmid transfer. *Genome Res* 2011, 21:477-486.
2. Aouida M, Page N, Leduc A, Peter M, Ramotar D: A genome-wide screen in *Saccharomyces cerevisiae* reveals altered transport as a mechanism of resistance to the anticancer drug bleomycin. *Cancer Res* 2004, 64:1102-1109.
3. Kitagawa T, Hoshida H, Akada R: Genome-wide analysis of cellular response to bacterial genotoxin CdtB in yeast. *Infect Immun* 2007, 75:1393-1402.
