# Supplementary material for: A Genomic Screen Revealing the Importance of Vesicular Trafficking Pathways in Genome Maintenance and Protection against Genotoxic Stress in Diploid Saccharomyces cerevisiae Cells
Source: PLoS One. 2015 Mar 10;10(3):e0120702. doi: 10.1371/journal.pone.0120702 (PMC4355298; doi:10.1371/journal.pone.0120702)
Supplement: S1 Table — (PDF) [file pone.0120702.s006.pdf]

S6 Table. Strains used in this study.

Strain	Genotype	Source
BY4741	*MAT*a *his3Δ1 leu2Δ0 met15Δ0 ura3Δ0*	Euroscarf
BY4742	*MAT*α *his3Δ1 leu2Δ0 lys2Δ0 ura3Δ0*	Euroscarf
BY4743	*MAT*a/*MATΔ his3Δ1/his3Δ1 leu2Δ0/leu2Δ0 LYS2/lys2Δ0 met15Δ0/MET15 ura3Δ0/ura3Δ0*	Euroscarf
1n (YAS281)	*MAT*a *his3Δ1 leu2Δ0 met15Δ0 URA3*	[1]
αC:L (YAS288)	*MAT*α *his3Δ1 leu2Δ0 lys2Δ0 ura3Δ0 can1::LEU2*	[1]
2n (YMS14)	*MAT*a/*MAT*α *his3Δ1/his3Δ1 leu2Δ0/leu2Δ0 LYS2/lys2Δ0 met15Δ0/MET15 URA3/ura3Δ0 CAN1/can1::LEU2*	[1]
1n *arf1* (YBI5)	*MATa his3Δ1 leu2Δ0 met15Δ0 URA3 arf1::kanMX4*	This work
αC:L *arf1* (YBI6)	*MATα his3Δ1 leu2Δ0 lysΔ0 ura3Δ0 can1::LEU2 arf1::kanMX4*	This work
2n *arf1/arf1* (YMS16)	*MATa/MATa his3Δ1/ his3Δ1; leu2Δ0/ leu2Δ0; LYS2/lys2Δ0; met15Δ0/ MET15; URA3/ura3Δ0 CAN1/can1::LEU2 arf1::kanMX4/arf1::kanMX4*	This work
1n *gga2* (YKK26)	*Mat a his3Δ1 leu2Δ0 met15Δ0 URA3 gga2::kanMX4*	This work
αC:L *gga2* (YKK27)	*MATα his3Δ1 leu2Δ0 lysΔ0 ura3Δ0 can1::LEU2 gga2::kanMX4*	This work
2n *gga2/gga2* (YMS24)	*MATa/MATa his3Δ1/ his3Δ1; leu2Δ0/ leu2Δ0; LYS2/lys2Δ0; met15Δ0/ MET15; URA3/ura3Δ0 CAN1/can1::LEU2 gga2::kanMX4/gga2::kanMX4*	This work
1n *nhx1* (YKK29)	*Mat a his3Δ1 leu2Δ0 met15Δ0 URA3 nhx1::kanMX4*	This work
αC:L *nhx1* (YKK28)	*MATα his3Δ1 leu2Δ0 lysΔ0 ura3Δ0 can1::LEU2 nhx1::kanMX4*	This work
2n *nhx1/nhx1* (YMS26)	*MATa/MATa his3Δ1/ his3Δ1; leu2Δ0/ leu2Δ0; LYS2/lys2Δ0; met15Δ0/ MET15; URA3/ura3Δ0 CAN1/can1::LEU2 nhx1::kanMX4/nhx1::kanMX4*	This work
1n *pep12* (YKK19)	*Mat a his3Δ1 leu2Δ0 met15Δ0 URA3 pep12::kanMX4*	This work
αC:L *pep12* (YKK20)	*MATα his3Δ1 leu2Δ0 lysΔ0 ura3Δ0 can1::LEU2 pep12::kanMX4*	This work
2n *pep12/pep12* (YMS18)	*MATa/MATa his3Δ1/ his3Δ1; leu2Δ0/ leu2Δ0; LYS2/lys2Δ0; met15Δ0/ MET15; URA3/ura3Δ0 CAN1/can1::LEU2 pep12::kanMX4/pep12::kanMX4*	This work
1n *rad52:H* (YAS315)	*Mat a his3Δ1 leu2Δ0 met15Δ0 URA3 rad52::HIS3*	This work
αC:L *rad52:H* (YAS314)	*MATα his3Δ1 leu2Δ0 lysΔ0 ura3Δ0 can1::LEU2 rad52::HIS3*	This work
2n *rad52:H/rad52:H* (YAS330)	*MATa/MATa his3Δ1/ his3Δ1; leu2Δ0/ leu2Δ0; LYS2/lys2Δ0; met15Δ0/ MET15; URA3/ura3Δ0 CAN1/can1::LEU2 rad52::HIS3/ rad52::HIS3*	This work
1n *vid22* (YKK21)	*Mat a his3Δ1 leu2Δ0 met15Δ0 URA3 vid22::kanMX4*	This work
αC:L *vid22* (YKK22)	*MATα his3Δ1 leu2Δ0 lysΔ0 ura3Δ0 can1::LEU2 vid22::kanMX4*	This work
2n *vid22/vid22* (YMS19)	*MATa/MATa his3Δ1/ his3Δ1; leu2Δ0/ leu2Δ0; LYS2/lys2Δ0; met15Δ0/ MET15; URA3/ura3Δ0 CAN1/can1::LEU2 vid22::kanMX4/vid22::kanMX4*	This work
1n *vps1* (YKK25)	*Mat a his3Δ1 leu2Δ0 met15Δ0 URA3 vps1::kanMX4*	This work
αC:L *vps1* (YKK18)	*MATα his3Δ1 leu2Δ0 lysΔ0 ura3Δ0 can1::LEU2 vps1::kanMX4*	This work
2n *vps1/vps1* (YMS21)	*MATa/MATa his3Δ1/ his3Δ1; leu2Δ0/ leu2Δ0; LYS2/lys2Δ0; met15Δ0/ MET15; URA3/ura3Δ0 CAN1/can1::LEU2 vps1::kanMX4/vps1::kanMX4*	This work
1n *vps3* (YBI8)	*Mat a his3Δ1 leu2Δ0 met15Δ0 URA3 vps3::kanMX4*	This work
αC:L *vps3* (YBI3)	*MATα his3Δ1 leu2Δ0 lysΔ0 ura3Δ0 can1::LEU2 vps3::kanMX4*	This work
2n *vps3/vps3* (YMS22)	*MATa/MATa his3Δ1/ his3Δ1; leu2Δ0/ leu2Δ0; LYS2/lys2Δ0; met15Δ0/ MET15; URA3/ura3Δ0 CAN1/can1::LEU2 vps3::kanMX4/vps3::kanMX4*	This work
1n *vps45* (YKK33)	*Mat a his3Δ1 leu2Δ0 met15Δ0 URA3 vps45::kanMX4*	This work
αC:L *vps45* (YKK34)	*MATα his3Δ1 leu2Δ0 lysΔ0 ura3Δ0 can1::LEU2 vps45::kanMX4*	This work
2n *vps45/vps45* (YMS30)	*MATa/MATa his3Δ1/ his3Δ1; leu2Δ0/ leu2Δ0; LYS2/lys2Δ0; met15Δ0/ MET15; URA3/ura3Δ0 CAN1/can1::LEU2*	This work

	vps45::kanMX4/vps45::kanMX4	
1n *vps51* (YKK30)	*Mat a his3Δ1 leu2Δ0 met15Δ0 URA3 vps51::kanMX4*	This work
αC:L *vps51* (YKK31)	*MATα his3Δ1 leu2Δ0 lysΔ0 ura3Δ0 can1::LEU2 vps51::kanMX4*	This work
2n *vps51/vps51* (YMS29)	*MATa/MATa his3Δ1/ his3Δ1; leu2Δ0/ leu2Δ0; LYS2/lys2Δ0; met15Δ0/ MET15; URA3/ura3Δ0 CAN1/can1::LEU2 vps51::kanMX4/vps51::kanMX4*	This work
1n *vps63* (YKK23)	*Mat a his3Δ1 leu2Δ0 met15Δ0 URA3 vps63::kanMX4*	This work
αC:L *vps63* (YKK24)	*MATα his3Δ1 leu2Δ0 lysΔ0 ura3Δ0 can1::LEU2 vps63::kanMX4*	This work
2n *vps63/vps63* (YMS20)	*MATa/MATa his3Δ1/ his3Δ1; leu2Δ0/ leu2Δ0; LYS2/lys2Δ0; met15Δ0/ MET15; URA3/ura3Δ0 CAN1/can1::LEU2 vps63::kanMX4/vps63::kanMX4*	This work

1. Alabrudzinska M, Skoneczny M, Skoneczna A (2011) Diploid-specific genome stability genes of *S. cerevisiae*: Genomic screen reveals haploidization as an escape from persisting DNA rearrangement stress. PLoS One 6(6): e21124.
