# Supplementary material for: A Genomic Screen Revealing the Importance of Vesicular Trafficking Pathways in Genome Maintenance and Protection against Genotoxic Stress in Diploid Saccharomyces cerevisiae Cells
Source: PLoS One. 2015 Mar 10;10(3):e0120702. doi: 10.1371/journal.pone.0120702 (PMC4355298; doi:10.1371/journal.pone.0120702)
Supplement: S2 Table — (PDF) [file pone.0120702.s007.pdf]

S7 Table. Primers used in this study.

Primer name	Primer sequence
Can1.up	5'-AGAGCTCGATACGAGATAAAGCACAAAT-3'
Can1.lw	5'-GGCTGCAGTGAAGATAACGAAAAATGAGT-3'
Ura3.up	5'-AATCTAGACGATGATAACAAACCGAAGT-3'
Ura3.lw	5'-GGATCCGGCTACACCAGAGATACATA-3'
Kan-c.up	5’-TGATTTTGATGACGAGCGTAAT-3’
Rad52.kup	5’-AAGAAAAGACGAAAAATATAGCGGCGGGCGGGTTACGCGA CCGGTATCGA TCGGTGATGACGGTGAAAAC-3’
Rad52.klw	5’-AATAAATAATGATGCAAATTTTTTATTTGTTTCGGCCAGGAA GCGTTTCA AAAAACTTGATTAGGGTGAT-3’
Rad52.up	5’-CTTGCCCTGTAATGTCCTTT-3’
Rad52.lw	5’-ATAATGAATCTAATCCTGAA-3’
His3c.up	5’-ATGGTACCAACGATGTTC-3’
Arf1.up	5’-TTCGTTGGATTTACAAGG-3’
Arf1.lw	5’-CGGTGGACCTGAGAGCGA-3’
Gga2.up	5’-TAGTTTTACCCAATCCGCTCT-3’
Gga2.lw	5’-TTCGTCCTCATCTCCCAA-3’
Nhx1.up	5’- CGCCATTGTGTATCCATTTA-3’
Nhx1.lw	5’- TCTCCTTTCTTGCCTTTTCA-3’
Pep12.up	5’-CCTCGCCATAGTGTATCC-3’
Pep12.lw	5’-GAAGCTGCTCTTTCTAGG-3’
Vid22.up	5’-CAACAGATGAAGGACCAG-3’
Vid22.lw	5’-CACCAAGGTCTCTTCCAG-3’
Vps1bis.up	5’-TACCCTATTGGCAGTTTC-3’
Vps1bis.lw	5’-ACCTTCTTCATATTGGAC-3’
Vps3.up	5’-AAGAAAAGTATCAACAGG-3’
Vps3.lw	5’-TTGAAGAAGACCCAGAAG-3’
Vps45.up	5’-AAGAAAAGTATCAACAGG-3’
Vps45.lw	5’-CAACAACGCAATCTCAACAA-3’
Vps51.up	5’-GAGGCGTATTTGCGGTGAGA -3’
Vps51.lw	5’-TAGATGGGCGATTGGACGAA-3’
Vps63.up	5’-AGGTACGAAAACAACTGA-3’
Vps63.lw	5’-GACGTTACTGAGAAATGG-3’
JB3323/U1	5’-GATGTCCACGAGGTCTCT-3’
JB3324/D1	5’-CGGTGTCGGTCTCGTAG-3’
JB3325/U2	5’-CGTACGCTGCAGGTCGAC-3’
JB3326/D2	5’-ATCGATGAATTCGAGCTCG-3’
JB3327	5’-Cy3 - GTCGACCTGCAGCGTACG-3’
JB3328	5’-Cy5 - GTCGACCTGCAGCGTACG-3’
JB3329	5’-Cy3 - CGAGCTCGAATTCATCGAT-3’
JB3330	5’-Cy5 - CGAGCTCGAATTCATCGAT-3’
JB3348	5’-Cy3 - GGATACACTGACCAGCTACGATGAT-3’
JB3391	5’-Cy5 - GGATACACTGACCAGCTACGATGAT-3’
